# Supplementary material for: Barriers to optimal AEFI surveillance and documentation in Nigeria: Findings from a qualitative survey
Source: PLOS Glob Public Health. 2023 Sep 8;3(9):e0001658. doi: 10.1371/journal.pgph.0001658 (PMC10490937; doi:10.1371/journal.pgph.0001658)
Supplement: S1 Data — (ZIP) [file pgph.0001658.s002.zip › Transcription- interviews/Director of Public Health.docx]

DIRECTOR of Public Health, Kebbi State

**Interviewer:** Are you aware of the CDC system evaluation attributes. Have you heard about it before?

**Participant:** Yes, I have heard about it

**Interviewer:** Ok, great!

**Interviewer:** Do you think the current AEFI surveillance system in the Nigeria, particularly with your experience in Kebbi, is simple, flexible, acceptable and sensitive enough to guide us when it comes to vaccine safety profile?

**Participant:** Yeah, actually it is very simple, it is very flexible. But, there are issues such as training problems, poor understanding from the workers who are working with the system. However, the AEFI surveillance system is very holistic and fully robust by design.

**Interviewer:** Do you think the system AEFI surveillance system on its own is simple, flexible, acceptable and sensitive enough but if you think there are challenges with the operationalization that makes it insensitive, please feel free to express your opinion/thoughts?

**Participant:** Exactly, it is very simple, it is very acceptable, very flexible but in terms of the sensitivity there are lots of issues because not all of the cases are being picked up at the lower level, i.e., health facility, and people need to be sensitized within the community so they can even report the AEFI by themselves without relying on the healthcare workers. In my own opinion, the sensitivity is diminishing- is not all that right.

**Interviewer:** I would like to also ask- do you think the data being generated from AEFI surveillance system is of high quality, useful and timely to inform vaccine safety consideration?

**Participant:** In terms of timeliness, there are some gaps because we pursue them before they send the data. Hence, the data does not come timely. Also, in terms of quality, there are some documentation issues. Some pieces of information were often missed, so the data being generated is not of high quality.

**Interviewer:** So, it could affect the vaccine safety consideration based on the above?

**Participant:** Yes, exactly

**Interviewer:** Is there any difference in reporting pattern from within the rural health facilities and the urban facilities? Where do you have somewhat better reporting-rural and urban health facilities?

**Participant:** Actually, the reporting system is almost the same in both rural and urban facilities, but the report is not as it is expected to be. It is very scanty, is too small.

**Interviewer:** What are the challenges or bottlenecks or impediments to optimal AEFI surveillance and documentation from the point of detection, reporting, investigation and even the use of the data?

**Participant:** So, let’s start from the grassroot in terms of reporting and documentation that is where the issues come from. The data passes through the middle level where the AEFI committee investigate and send to the national for causality assessment. All experts are there at the national level to conduct causality assessment- that is to establish a relationship between the vaccination, vaccine itself and that illness or sickness that is caused or whether there is no causal relationship. But the major problem is at the grassroot level where the data is not being generated as it is supposed to, the expected number of cases are not being picked accordingly. Most of the staff at the health facility level want financial incentive before they will do their jobs. Thirdly, time factor-because some people are loaded with so many activities in the health facilities. This is because there is a maldistribution of human resources at the facility level. For example, you can get to a facility, where 40 people are assigned as staff, and in another, only one person is functioning as the RI and surveillance officer- the lone person is doing everything in the facility. So, the workload affects reporting. The has implication of the capacity of the system to conduct causality assessment because of paucity and quality of data.

**Interview:** Any other challenges apart from funding, maldistribution of health workers, work overload, lack of financial motivation. what other challenges?

**Participant:** Also, lack of accountability affected the AEFI surveillance system because actions are not taken against any staff for misconduct in the discharge of their duties in terms of managing funds, in terms of doing the right work at the right time, and the quality of data being generated.

**Interviewer:** Any other one?

**Participant:** Also, lack of supervision affected the quality of AEFI surveillance and documentation because when the personnel are not being supervised, they often do not do the right thing as it is supposed to be. But when there is proper supportive supervision, there is will be corrections and better output. Apart from supportive supervision, there is lack of prioritisation of AEFI. It is not given priority attention even to capture some cases in event-based surveillance system. AEFI surveillance has not been practically linked to event-based surveillance, which is essentially driven by alerts from rumour.

**Interviewer:** What is your perception regarding the functionality of AEFI surveillance and documentation for routine immunisation compared to supplementary immunisation activities or outbreak response (OBR). What is your perception, what do think about the functionality-are there differences?

**Participant:** There are lot of differences between the AEFI in RI and SIAs. First, the robust funding for SIAs- a lot of money is made available for the SIA activities while less money is made available to routine immunization. Routine immunisation service is part of routine duties of the health workers and therie is no additional financial motivation. Also, lack of supervision affected in AEFI surveillance and documentation for routine immunisation. However, in SIAs, there is a lot of supervision from partners and government within the short period of time. But RI is on weekly basis as scheduled and is continuous and the level of supervision is very low. Therefore, inadequate finance, inadequate supervision and lack of prioritisation- AEFI surveillance and documentation is not given high priority attention. As we strive to control and eradicate vaccine-preventable diseases, we must continue to strengthen routine immunisation across different age groups, we must also prioritise AEFI surveillance.

**Interviewer:** What do you think about AEFI reporting and documentation at the facility level? How do you describe the situation of AEFI reporting and documentation at the facility level?

**Participant:** The documentation and reporting of AEFI is very poor. The system needs to pick all the AEFI cases with good quality data (documentation) so that the investigators and evaluators can be able to ascertain or judge whether there is a causal relationship between the vaccine and the diseases

**Interviewer:** Do you think this information we are getting from the health facilities is ample-good enough to make causality assessment?

**Participant:** No, is not good, because we need more information, we need to pick up more cases, then we can assess causality.

**Interviewer:** At the LGA level, how will you describe the flow of data and the linkages from facility to the LGA and then into data management system like DHIS2 and IDSR003?

**Participants:** The IDSR data is from the focal person to the LGA DSNO on hard copy and now there is electronic copy transmitted to the server. Then, the DSNO & State Epidemiologist can have access to it. But in the DHIS, it is the LGA M&E officer that collates data at the health facility level send to the DHIS2. The M&E officer has access, the state DSNO has access and the State Epidemiologist has access to the DHIS data. Sadly, AEFI data is not being captured neither in the IDSR, nor on the DHIS. It is good if it is integrated into DHIS and IDSR.

**Interviewer:** But there is gross under-reporting based on available data?

**Participant:** It is under-reported seriously. The reported data from the health facility is minimal compared to the unreported cases in the field (communities)

**Interviewer:** How would you describe the LGA level data linkage with the existing data management system particularly IDSR and DHIS2 in terms of timeliness and completeness

**Participant:** It is very poor. There is gross under-reporting. Something needs to be done to improve AEFI surveillance system

**Interviewer:** What would you recommend to improve the AEFI surveillance and documentation in Kebbi State, and Nigeria, as a whole?

**Participant:** The first is to improve on the training and the retraining

**Interviewer:** How frequently do you think they should be conducting this training?

**Participant:** The training needs to be on a quarterly basis so that (health workers) people will not forget**.** Secondly, supervision should be conducted at the LGA and facility level- from the national down to the facility level.

**Interviewer:** What else apart from training, supervision?

**Participant:** Training, supervision and motivation and logistics

**Interviewer:** Motivation, how and which kind of logistics are you referring to?

**Participant:** Logistics- materials that are used to capture the data, i.e., the data tools.

**Interviewer:** So, which kind of motivation are you referring to?

**Participant:** To improve on the incentives, i.e., incentives for those people that works, not for everybody

**Interviewer:** Anything else?

**Participant:** Also, the AEFI community informants need to be revived, and there is need for intensive and high quality social and behaviour communication change because people need to get aware, people need to be informed at the community level so that they can they report themselves without relying on the healthcare facility reporting. Most of the cases are being picked by some health care workers, implying that there are many pending or unreported cases that are relevant to make causality assessment. This helps to establish whether there is a causal relationship between the vaccine and the sickness. Also, there is a lack of AEFI kits for routine immunisation(RI) service because only during SIAs will you see kits. During RI, kits are not available at the facility. So, there is a need to provide AEFI kits for routine service to manage adverse events. Also, with social and risk communication, people need to be aware of AEFI so that they can report many cases. In general, the way it is being operated in Kebbi State and Nigeria at large, it is sub-optimal. Therefore, it cannot pick up all the cases and may not be robust enough to guide vaccine safety in this country because of the stated challenges that need to be addressed.

**Interviewer:**

Thank you very much, the Director of Public Health. I am very grateful for this opportunity and your time and Insha Allah, God willing, we will be able to use this information to provide evidence for policy improvement and practice as regards the AEFI surveillance and documentation in Nigeria. Thank you once again

Participant:

Thank you
