# Supplementary material for: Barriers to optimal AEFI surveillance and documentation in Nigeria: Findings from a qualitative survey
Source: PLOS Glob Public Health. 2023 Sep 8;3(9):e0001658. doi: 10.1371/journal.pgph.0001658 (PMC10490937; doi:10.1371/journal.pgph.0001658)
Supplement: S1 Data — (ZIP) [file pgph.0001658.s002.zip › Transcription- interviews/Interview with NAFDAC Pharmacovigilance NFP.docx_transcribed.docx]

Interviewer: Introduction done, consents taken, and ethical principles of good research adhered

Participants: Okay, that is fine. Go ahead. Thank you very much ma.

Interviewer: Given your experience as the focal point for pharmacovigilance in Nigeria, I would like to know whether you feel the current surveillance system in Nigeria is simple, flexible, and acceptable and sensitive enough to ensure vaccine safety consideration?

Participant: The AEFI in Nigeria, based on paper, is okay, but looking at the operationalization of the system and how much data it is able to capture, I think that there is still a lot of gap to be filled and a lot of manpower capacity building, a lot of attitudinal changes in terms of healthcare workers’ knowledge, attitude and practice with respect to AEFI needs to be remodelled.

Interview: So, you think as it is operationally, it is not sensitive enough to inform vaccine safety consideration?

Participant: It is not sensitive enough to track the AEFI that are occurring within the system.

Interview: Do you think the data being generated from the AEFI surveillance system is of high quality and useful, as well as timely to inform vaccine safety consideration, operationally?

**Participant:** Currently, I will say no, because we are seeing a sense of timeliness of reporting and for serious AEFI, timeliness of investigation and transmission of report where it could be used for informed decision making becomes inadequate. We begin to see within the routine system of the vaccine operations in-country, the reporting of AEFI has not been well entrenched in the system. Reason being that they feel they are minor AEFI that can just be treated without actually reporting it. The data on minor AEFI are not transmitted up, then even with the occurrence of those serious AEFI which are usually seen during campaign, the investigation reports are not properly done and not complete. So, you begin to see problem. We have the national expert committee that actually does causality assessment for serious AEFI who are supposed to be meeting quarterly. But because of inadequate reporting, it becomes very very difficult to bring them together to do some serious causality assessment on the report. Without report, you cannot actually call for a meeting. So, I think in terms of timeliness of reporting and transmission of report and in terms of the quality of report even when investigations are done, they send up investigation form without the report form, so the report is often incomplete to do any adequate causality assessment.

**Interviewer:** In summary, will you that the current AEFI surveillance system as it is being operated is effective and robust to inform vaccine safety consideration and communication strategy to drive demand for immunisation?

**Participant:** The current system is effective to drive demand for vaccination but driving demand to drive AEFI reporting is not the case. But because of inadequacies in terms of reports transmission and delays in transmitting of report, at the national level, we have done some gap analysis and we have found out that there is need to bridge the gap in terms of reporting. Now there is an app called Med Safety app which is a handheld device. The AEFI forms are put on a software called the Med Safety app which can be operated using the mobile phone, android, phones that are using the IOS system. People can easily go to playstore on IOS and download the Med Safety App. So, what we did is inputted the form into the Med safety app, i.e., AEFI reporting form into Med Safety app. Initially it was designed for ADR (adverse drug reaction) but we have seen that there is so much gap in terms of AEFI reporting, so the hardcopy form that are being used for reporting AEFI are now uploaded on the Med Safety App. Over 60% of the Nigerian adult populace has access to use android or iPhone, so they can easily download the app. Once there is an AEFI report anywhere, all they need to do is just click on the icon for the Med Safety and bring out the form. And immediately you report and submit the information, it goes to the national agency direct- real time. It makes it easier for people and agency to actually make informed regulatory decision, to inform pattern and the types of AEFI that was seen with the different vaccine component. Now we actually piloted it with the COVID-19 vaccine because there is a project, African Union Smart Safety Surveillance that is Nigeria is part of. The COVID-19 vaccine was used as a pilot to actually incorporate the form (software app) widely into the system that people can actually use it. This is the first term in the history of a campaign that we are getting within the spate of time in a month we are getting up to 1800 AEFI reports uploaded directly and correctly. We see it and make statistical analysis and clustering, the pattern, what is happening in the field, serious and non-serious AEFI. For a novel vaccine, you need to capture whether serious or non-serious because the complete safety profile of the vaccine is not completely known. So, by capturing the serious and non-serious AEFI, it provides a safety profile for our own people because clinical trials were not actually done within our own population for the COVID-19 vaccines. The Med Safety app is providing an opportunity to bridge the gap of poor reporting and inadequate reporting.

Interviewer: What are the challenges of AEFI surveillance and documentation in Nigeria, based on your working experience?

**Participants:** We have been able to find out form studies that the reasons why people are not reporting mostly is sometimes poor knowledge what needs to be actually reported, and the fear of reprimanding- feeling that the cause of the AEFI is actually as a result of inadequacies in their professional practice, which is wrong. …and attitudinal changes will tackle that particular component because it is a barrier. Another barrier that we have identified is people feeling it is just AEFI, once we treat there is no need to report. It also has to do with attitudinal change and capacity building. They need to understand the reasons why they need to report because it is their professional obligation to the patient or to the vaccinee. Then, another challenge that we are seeing, we tend to have the AEFI committee within the LGA and the State, who are actually supposed to take responsibilities of monitoring and investigating serious AEFI. Because there are inadequate logistics provision for them to hold regular meetings, carry out their functions as maybe required, these meetings are barely done within LGA and State except during campaigns because funds are provided during campaign. AEFI monitoring and reporting should be an integral part of routine immunisation system in Nigeria. This is important, even when campaign comes it makes it easier as there is already a process or a procedure that is ongoing system. It makes it easier for ease of reporting, ease of transmitting and . As long as AEFI surveillance system is not an integral part of the routine immunisation system, this gap will always exist. Then, issues that also have to do with logistics, for example, if somebody comes to the health facility, get immunized and the person is from hard-to-reach area (HTR) of the LGA, the health care workers will need some logistics support for adequate investigation and reporting. Once the logistics support is not available for the health care workers to do that work adequately, it will never be done. These are some of the bottlenecks within the system that need to be strengthened.

**Interviewer:** Why is the functionality of AEFI surveillance and documentation for outbreak response/supplementary immunisation activities seems better compared to routine immunisation?

**Participant:** The answer is very simple. Within campaign, there is a lot of funds provided to ensure that the campaign is carried out. And the AEFI component in terms of monitoring is also integrated into the campaign. Money is flowing and people are paid within that time to do that specific work within that timeline.

**Interviewer:** Do you think the AEFI reporting and documentation at the HF or LGA level effectively feed into data management platforms like IDSR, DHIS 2, SORMAS? How does it integrate into the Med Safety app?

**Participant:** The IDSR with the DHIS2 tool, I know there is also SORMAS, they look at AEFI as an aggregated number and not in terms of meeting the key indicator in the reporting of AEFI. So, when they are reporting they are reporting in the sense of this month.. we have about 30,000 AEFI reported. What is the breakdown of that 30,000, what types of AEFI are they, who is the patient that this has happened to? You cannot make any important deduction or statistical analysis from the report generated from IDSR or DHIS 2 or SORMAS and all that. Right now, there are plans, works are ongoing to really actually narrow into the AEFI reporting format to the different variable in the form so that actually when you say you have two AEFI reports, you can pull out that report, it has happened to a patient, who is the patient, what type AEFI are we talking about, what was the person given, what kind of vaccine, what other vaccines were given along the suspect vaccine, was there other concomitant disease treatment that are being given to the patient? Because all these things will aid in the causality assessment for that particular AEFI report. So, that is the inadequacy of the IDSR in term of AEFI. Actions are ongoing, the DHIS 2 from NPHCDA point of view, they are expanding the DHIS2 to capture specific AEFI one after the other, not in terms of numbers. What is currently being presented, for me, because they presented the work they are doing in term of reporting. We should try as much as possible to remove bottleneck in term of reporting. We already have a lot of bottlenecks. For the DHIS 2 AEFI reporting platform I saw them building also already is coming out with a lot of bottleneck. Because form the platform they have developed, the HF will report, when the facility reported, the DSNO at the LGA will have to authenticate the report first, from the LGA, it will move to the State to authenticate the report. You know our system, you can build castles in the air, but it is the implementation that is actually very very key. It is the same DSNO is reporting on all disease surveillance in the LGA and is the same DSNO that will look at the different AEFI from all the health facilities within the LGA before the report can be transmitted up. I don’t think it will be very functional and effective when it is operational. That is actually why the Med Safety App is made in such a way that whether you are healthcare provider or a consumer (public), once you are literate to operate the app, it is simple. Give us what your name is, what vaccine did you receive, what is the AEFI we are talking about? The only thing we need to do is make the system interoperable- fitting into each other. Not until that is done, the Med Safety app is the most informative for now. All the COVID-19 vaccine safety reports to NPHCDA from States are mere numbers, e.g., we have 3000 non-serious, 1000 non-serious- it is just number! The line list for the COVID-19 vaccine safety is not readily available from States. The only report the country can boast of in the country in terms of vaccine safety for COVID-19 is what is already uploaded on the Med Safety app. That is the only information the country has now. And NPHCDA has seen that that is the most operational one now, they are now calling back to the State to say that those numbers of AEFI that you have given to us, please go and upload it. Your State Epidemiologist and State DSNO should upload on the Med Safety app because (that is) when they now ask the country provide your evidence since NPHCDA is saying (for example) they have 10,000 or 13,000 AEFI reported for COVID but the only thing that is uploaded on the Med Safety app is about 5000, and that is the only 5000 you can see the complete report for the patient/vaccinee. So, you see what we are saying and from that report, it is easier for the country to say in terms of AEFI, more of headache is being reported, more of anaphylaxis in terms of AEFI is being reported, but giving numbers, we have 10,000, we have 2,000, where are the reports? These are the bottlenecks in system operations within the country.

Interviewer: Is there a way the leadership of NAFDAC can advocate, given this argument that you have presented- they are quite correct and top-notch?

Participant: No! Now, we have linked the leadership of NAFDAC and NPHCDA. And it is confidential, because we have made presentation to them and we argued about what your (current) system is saying, bring out the report, this is what our system has generated, these are the reports. It is patient (vaccinee) specific. That argument had been done at that level and they have understood it, and that is why they have called back to the State Epid and State DSNOs, that all the reports they have sent to them (NPHCDA) should be uploaded on Med Safety app.

Interviewer: How do they intend to institutionalise the use of Med Safety app such that all the health workers and individual within the communities would have it on their phone (for reporting) without having to distort current system to achieving a qualitative data generation that would be useful for causality assessment?

**Participant:** Yes, the Med Safety app is not just a NAFDAC app now because it was launched by the Minister of Health in November last year (2020). And all stakeholders such as NPCHDA, NAFDAC and others were in the launch. It is not a NAFDAC, it is a Nigerian app for capturing AEFI data. A lot of capacity building are ongoing. You know transiting from paper to e-reporting, there should be a transition, it is not something everybody keys in at once. But at least, everybody opportunity that present itself, Med Safety App can be presented. And you as a WHO State Coordinator, these are the things you need to bring to your State. What do we know about the Med Safety app? You already know it. It is for you to introduce during the State level meetings so that the people will know that this something they have to continue to use because it provides evidence-based report, well-timed. And the Med Safety app is done in such a way that NAFDAC and NPHCDA have access to it. It transcends the State. Both NPHCDA and NAFDAC Chief Executives at that high level have agreed that (Med Safety App)it is the way to go and it has been presented before the Minister. And the Minister had asked the two (agencies leadership) to go back and finalise the reports into one app. That they should go and (integrate) streamline the two recommendational reports.

Interview: Based on your expertise as the National Focal Point for Vaccine Safety and Pharmacovigilance and given your experience visiting Kebbi State, what are your recommendations to improve AEFI surveillance and documentation in Nigeria?

Participant: First and foremost, it is everybody’s work, not one person’s work. There has to be opportunities for capacity building for health workers. Secondly, if there is a way to make the AEFI committee very functional, that is also important. Attitudinal changes- people need to understand the purpose, the need, the importance of AEFI reporting and not business as usual. And there has to be some logistics provision for AEFI monitoring and strengthening, that is very important.

Interviewer: How do we raise community awareness, especially with the use of Med Safety App, and their capacity to report?

Participant: Awareness creation and capacity building are very key. If there is a way community sensitization and mobilisation can be done, that will help a lot that. When people begin to understand that we have put the reporting even in their hand through the use of their mobile phones. And the beauty of the Med safety app is such that once you have downloaded it on your phone and you are doing a report, you don’t really need data, the only time you need data is when you have completed the form and you want to submit. Just put on your data and submit.

Interviewer: If there are issues at the State and LGA level, are there funding and coordination gaps at the national level as well?

**Participant:** At the national level now, for the AEFI committee at the national level, that is currently being funded by WHO, bringing them together for causality assessment. That is not a problem because that one is functional, but their work is determined or dependent on the report generated from the locality (LGA level).

**Interviewer:** If we are to direct advocacy, at what level will you recommend that we direct advocacy given the current effort to institutionalise Med Safety app and keeping in mind gaps seen in the current/existing system and the quality data being generated from Med Safety app?

**Participant:** Government need to do that too (show commitment). Because everywhere, globally now, there is a global shift to pharmacovigilance, anybody giving you money is saying pharmacovigilance is very key. You cannot be providing vaccine without determining the safety of that vaccine. Government is supposed to be doing their own bit, partners are also supposed to be doing their own bit. People providing us with vaccines should make provision for logistics for AEFI monitoring. It is very critical. It is a *sine qua non.*

**Interview:** That is a lesson I have learnt from this discussion, that even the manufacturers have a role to play in AEFI monitoring?

**Participant:** Yes, they have a role to play. You cannot just provide vaccine without providing logistic support for safety monitoring. It is just because in public health programme, we grant them waiver because they are donated vaccines. For marketing organization holders, those that register vaccines in-country, it is mandatory for them to have a pharmacovigilance system because once you see safety issue that the risk is higher than the benefit. Obviously, we mop out the vaccines from circulation, whether you bring them in thousands or millions it doesn’t matter. You must have a system. And that is why for public health programme when we give them waiver to bring these things in, we hold the national programme responsible for AEFI safety monitoring. But what we are only doing is that we are giving you waiver to bring these vaccines, it is your responsibility to have your system in place to ensure since you are giving en masse to people, to ensure the vaccine is safe among the target population.

Interview: Thanks for the opportunity.
