# Supplementary material for: Barriers to optimal AEFI surveillance and documentation in Nigeria: Findings from a qualitative survey
Source: PLOS Glob Public Health. 2023 Sep 8;3(9):e0001658. doi: 10.1371/journal.pgph.0001658 (PMC10490937; doi:10.1371/journal.pgph.0001658)
Supplement: S1 Data — (ZIP) [file pgph.0001658.s002.zip › Transcription- interviews/Interviewer_NAFDAC-Kebbi.docx]

Interviewer: Are you comfortable to proceed

Participant: Yes of course

Interviewer: When talking, you don't need to mention your name. it's not necessary. Basically, I would like to know, first of all are you aware of the CDC surveillance system evaluation attributes have you ever had anything about it? CDC's surveillance system evaluation attributes

Interviewer: Do you know about it?

Participant: I know about it

Interviewer: Ok good. So anyway, there are a number of attributes that one can use to assess a public health surveillance system. For the purpose of this study, we are assessing the AEFI surveillance system through attributes such as the simplicity- how simple is the surveillance system in terms of his operation, is it flexible, can it easily be adapted, can it allow updating of information about cases that are being reported, can it be integrated into another system, is it acceptable to all stakeholders, NAFDAC, NPHCDA, WHO, health workers in the field and is it sensitive enough to inform vaccine safety consideration? So, those are the attributes that we are trying to use to define the surveillance system, so I would like you to provide your own opinion on some of these attributes and if you do not have sufficient experience with it you can let us also know. So, what is your thoughts about it

Interviewer: The first one- simplicity, do you think the current AEFI surveillance system in Nigeria is simple to use?

Participant: Yes, it is very simple to use because I know last week when I was in the office you know already before we are using yellow forms. But the issue of yellow forms is outdated so we brought this AEFI app now and it's simple and is applicable to everyone, everyone can use it and you can download it through play store, you can go to play store and download it.

Interviewer: And that's medsafety app

Participant: Yes, that's medsafety app

Interviewer: So, the medsafety app to the best of my knowledge is a new application that has been introduced by NAFDAC

Participant: Yes

Interviewer: Good. But it has not really reached the operational level. It's still being piloted using the covid-19 vaccine AEFI surveillance. But like you said, I'm aware that talks are ongoing to actually use it at all level and for all AEFI. So, I quite agree with you that it's simple but do you also think that the paper one is simple?

Participant: But the AEFI is simple. What I say the paper one is simple

Interviewer: The paper documentation process

Participant: Because you know some people, they use to misplace the paper one. I can remember last time, you know we are using the paper one the yellow form, we use to visit some hospitals, some health facilities to distribute the yellow forms. But sometimes, when we get them these yellow forms, to return the yellow forms, you find out that is very difficult because they cannot it bring all these yellow forms especially those medical doctors, they are not willing to fill these yellow forms

Interviewer: So, that means your data collection system from health institution about vaccine safety is different, you have your own forms that you are using even before now

Participant: Yes

Interviewer: Even before the introduction of the medsafety app

Participant: Yes

Interviewer: Ok, that's fine. But do you think the system is flexible, acceptable and sensitive enough, do you think it can pick all AEFI cases in the population the way it is being operated

Participant: Yes, well what I can say for now, I think it's flexible because I know I have participated on this COVID-19 vaccine recently

Interviewer: Ok

Participant: And I have collected all the AEFI cases in malaria control and I have uploaded all of them into the medsafety app and I think is flexible

Interviewer: Ok. Good, do you think is acceptable to all stakeholders

Participant: All stakeholders like

Interviewer: Including your organization, do you think the way we are running it now, I mean outside even the newly introduced medsafety which has not been totally in use, the system prior to the introduction, the piloting of medsafety do you think that system is acceptable to everybody?

Participant: Yes, what I can say here it is acceptable because what I said earlier last month or last two months, we received a courtesy call visit from medical doctors from FMC they came to our office they said that they want to know the issue about these AEFI because they are aware of it but they don't know how to operate it, so with my boss we sat down and we showed them how to operate it. They said they are even happy, therefore this one is acceptable than the paper one.

Interviewer: So, now the medsafety app being introduced is acceptable more than the yellow form (paper form being exclusively used by NAFDAC).

Interviewer: So, now, I think what I can say here is that the data being generated from IDSR and DHIS 2 is actually not being used, it doesn't really reach NAFDAC, NAFDAC has its own data collection tool. Is that what you trying to tell me?

Participant: Yes, I don't think we are using DHIS

Interviewer: You are not using DHIS, you are not using IDSR.

Participant: No.

Interviewer: So, you have your own data collection tool which is the yellow form you are referring to. To collect information about vaccine safety.

Participant: Yes, That's it

Participant: I only know about DHIS when I participated in COVID-19 vaccine roll-out.

Interviewer: First time you are hearing it?

Participant: First time I'm hearing the DHIS because I worked as a supervisor in Augie local government. So, I have downloaded it on my phone and I use it and is very interesting. But we in NAFDAC I don't think we are using DHIS

Interviewer: So, that means there is a problem with integration and there is a likely problem of under-reporting as well with this information you have provided

Participant: Yes

Interviewer: Because you won't get all the cases, you won't get all the cases because you even said that most of the time you find it difficult to get information from these people and do you give the yellow form to all the RI provider facilities in the state

Participant: Yes, we give

Interviewer: All the 661 health facilities

Participant: But you know we are not many here in NAFDAC

Interviewer: So, you are not getting information from all the facilities

Participant: We are not getting information

Interviewer: So that means the system as it is now is not sensitive enough. Do you agree with me?

Participant: Yes, in that way I can say yes. Because sometimes even when we have distributed the yellow forms sometimes, they don't report back

Interviewer: They don't return it

Participant: They don't give us back

Interviewer: But, that's why I'm saying that means there is drastic under-reporting

Participant: Yes

Interviewer: Then, do you think the little information being generated is enough to make a conclusion on vaccine safety

Participant: Is it on AEFI

Interviewer: AEFI, that AEFI Adverse event following immunisation the little data that is being generated, do you think is sufficient for you to say that we have enough to guide vaccine safety

Participant: Actually, it's not, because they are not many

Interviewer: Exactly, so that means the system is not effective and robust enough

Participant: Yes

Interviewer: Now, what are the challenges impeding optimal surveillance and documentation of AEFI based on your experience working in Nigeria and particularly in Kebbi state?

Participant: I told you earlier the challenges we have, you know this, there are some medical doctors that they are not willing to fill this AEFI

Participant: At health facilities, they are not willing to fill this AEFI

Interviewer: Ok

Participant: And I have participated in this COVID-19 vaccine and we showed them how to use this AEFI but they are not willing to fill this AEFI , they want me to come and fill it by myself, that's the challenges

Interviewer: What other challenges apart from poor workers attitude. So, what are the other challenges that you would like to talk about?

Participant: Yes, the other challenges I think we have is inadequate supervision by some of the regulatory agencies just like NAFDAC and National Primary Healthcare Development Agency and State Primary Health care Agency because of these human resources deficit. Then, the second one is that some of the health workers don't know the value of this AEFI that's why they are not filling these AEFI forms. Lastly, i think the government places less priority on the AEFI compared to disease surveillance.

Interviewer: Do you think the AEFI reporting and documentation at the facility level and LGA level feed in to IDSR or DHIS 2 or other AEFI data management platforms, do you think it effectively feeds in to IDSR and DHIS or any other AEFI data management platform?

Participant: Yes, what I see here is, I think there is problem. Because last week I went to malaria unit (SMoH) they called me from Abuja that I should go to malaria unit (SMoH)and check. All the cases have they uploaded it on AEFI

Interviewer: Have they uploaded them on what, on which platform

Participant: On medsafety app, so what I observe is I met one of my friends, I think I finished the same university with him he is a DSNO what i observe is they all line listed the cases. But the problem they don't upload all the cases. What they are waiting for, they are waiting for NAFDAC staff to come and upload it and they know how to do it. Is just that maybe, i don't know, they have several works or any other challenges, i don't know because these are part of challenges we have

Interviewer: So, as it is, it's not effectively feeding into the medsafety application

Participant: It's not effective

Interviewer: Is the information and data being generated of good quality?

Participant: From my experience, I think it's of good quality

Interviewer: Are you sure

Participant: Yes

Interviewer: But that's for covid, the one you are talking about, so you can't use covid to generalize

Participant: That's only for covid

Interviewer: For COVID-19, you can say that is because there is money there to support it but I'm talking about general experience

Participant: No

Interviewer: How can you describe it, the quality how will you describe it- is it good, poor or fair?

Participant: it's fair

Interviewer: And then in terms of timelines and completeness, is the data timely and complete generally not COVID-19 alone?

Participant: No, it's not timely, it's not complete

Interviewer: Then, what are your recommendations to improve AEFI surveillance in Kebbi state and Nigeria?

Participant: i think if we can conduct trainings or workshops for these health facilities and DSNO and the state primary healthcare even NAFDAC at the state level

Interviewer: How often do you think these should be conducted?

Participant: I think monthly or quarterly

Interviewer: What other thing apart from training do you think we need to do

Participant: Sensitisation of all stakeholders

Interviewer: What about moving from paper to the use of electronic, don't you think that is also important

Participant: It's important

Interviewer: Meaning we should digitalize

Participant: Yes, because what i say is important, most of the people now they are not good in paper they are good in electronic data collection

Interviewer: So we should consider the introduction of the electronic data collection at the health facility level and then data can be transmitted. How about at the community level do you think the awareness is good? What do you think we should do? Don't you think we should do something about that

Participant: Yes, at the health facility and at the community level

Participant: I think we can maybe, even in the community level i think we can do the sensitization and conduct some trainings or workshops

Interviewer: It's alright. Any other thing you want to say about recommendations is that all? On the part of NAFDAC what do you think can be done to support AEFI, data collection, improve data collection

Participant: What I will say have already said it. I think the thing we are supposed to do- I think we suppose to conduct some workshop and trainings for these health facilities people because they cannot do it alone, I think with NAFDAC, State Primary Health Care Development Agency, National Primary Health Care Development Agency, I think we can come together so that we can do sensitizations, workshops for those people.

Interviewer: It's alright, thank you very much. I'm very grateful. Thanks for the time and efforts.
