# Supplementary material for: Barriers to optimal AEFI surveillance and documentation in Nigeria: Findings from a qualitative survey
Source: PLOS Glob Public Health. 2023 Sep 8;3(9):e0001658. doi: 10.1371/journal.pgph.0001658 (PMC10490937; doi:10.1371/journal.pgph.0001658)
Supplement: S1 Data — (ZIP) [file pgph.0001658.s002.zip › Transcription- interviews/LGAF KEBBI.docx]

Interviewer: I hereby seek verbal consent for me to proceed with this interview

Participant: You can proceed

Interviewer: Thank you. So, basically I would like to first of all assess, I would you like to provide information on the surveillance system capacity and its functionality, using the CDC system attributes. Are you aware of the CDC system evaluation attributes?

Participant: Yes

Interviewer: Ok, do you think the AEFI surveillance system in Nigeria is simple, flexible, acceptable and sensitive enough to inform vaccine safety consideration. If so, can you please elaborate.

Participant: Honestly, the surveillance system is actually simple, but the flexibility is not actually something to write home about. There are issues associated with the flexibility of the AEFI surveillance system particularly in Kebbi State, based on my experience in the LGAs that I have worked.

Interviewer: What about the area of acceptability of the AEFI surveillance system and its sensitivity? Can you also describe it?

Participant: Yes, it is acceptable, but the sensitivity is not actually enough because it cannot detect all the suspected AEFI cases that are supposed to be reported.

Interviewer: Do you think the data being generated by the AEFI surveillance system or how would you describe the data being generated from the AEFI surveillance system in terms of quality, usefulness and timeliness to inform vaccine safety consideration?

Participant: Honestly, the data generated across or let me say the primary data source as far as AEFI surveillance is concerned in the health facility level and community level. So, you found that sometimes the service providers don't actually fill the data they are expected to fill. Sometimes, there used to be a delay in terms of transmitting the data from the health facility level to the next level, that is, the DSNOs who serve as the LGA responsible officer as far as AEFI surveillance system is concern

Interviewer: Thank you. Can you repeat you said about quality?

Participant: I said the quality is not, the AEFI data generated at health level is mostly not of high quality. You will found that there are lot of discrepancies with the way and manner even the forms are filled. Some will fill forms without the health information like you will found that somebody will be filling AEFI report only carrying the name of one person without accompanying the surname or may be the age is not filled or may be the some of the information about the vaccine are not also captured in the AEFI reporting form. Some may even submit only the reporting form without accompany it with line list while you will see some, at the end of the day they will only submit the line list that has summary without carrying primary reporting from of the AEFI

Interviewer: Thank you very much. Based on these attributes that you have discussed the overall. Do you think the current AEFI in Kebbi State and Nigeria, as a whole, is effectively and robust enough to inform vaccine safety consideration as well as to generate demand for immunization?

Participant: Honestly, it is not. Because had it been, let say the service providers, the system on itself can be able to detect all expected cases then may be after cases are reported they can be investigated there will be a timely feedback that will help ...

Interviewer: Thank you very much. How about the regarding the stability and representativeness of the surveillance system. Do you think it is representative and stable. Stable in terms of its reliability, that is ability to collect and manage and provide data properly without failure and then availability- ability to be operational when it is needed. So, how do you describe those two entities. Those two attributes.

Participant: Like the first one you said

Interviewer: Representative, whether it accurately describes the occurrence of AEFI and its distribution in the population.

Participant: Honestly, it is not for this because you found out that across the state, it is not all the facilities that are RI providing facility. Then also, it is not only RI providing (service provider) that are supposed to be reporting AEFI. Communities are not well sensitized unless during SIAs about AEFI. So, with this you found that a lot of cases that are supposed to be reported. Overall, the AEFI data that you have is not the true representative of what it supposed to be obtainable on ground

Interviewer: Thank you very much. How about stability. Do you think is stable?

Participant: Honestly it is not stable.

Interviewer: Can you describe, can you elaborate?

Participant: For it to be stable, it the sensitivity has to be very high

Interviewer: Is alright. Thank you. But how about as a subset of the stability there is also the component of availability. Do you think it is available?

Participant: Yes, it is available of course because they have reporting forms, they have and sometimes there are trainings also.

Interviewer: Is alright and the system is in place most of the time. As regards the challenges or the bottlenecks impeding optimal AEFI surveillance and documentation in Nigeria, based on your own experience? What are those challenges, and can you elaborate on them?

Participant: Honestly, to me, the first challenge is no much priority is given to AEFI surveillance as far as government is concerned. You found that most of the activity associated with AEFI are sponsored by partners. Then, also the service providers are not well knowledgeable about the case definition sometimes or even what to report even these cases are detected. And then, there are no specific responsible officers at the LGA level, at state level, there are no focal person that are responsible for collation or analysing AEFI data I think at LGA and health facility level, unless if responsibilities are attached to the DSNO -the surveillance officer.

Interviewer: What else?

Participant: Challenges, there is also logistic challenges. You found that AEFI surveillance is not funded by government. No funding from government going to that aspect. And there are no provisions of management drugs, even AEFI kits, based on my experience. In all the LGAs I have worked, hardly you go health facility that is conducting RI and they have AEFI kit, if you see any AEFI kit available in the health facility that may be one provided during SIAs campaign (injectable SIAs campaign)

Interviewer: Ok. What about the, what is your experience, what are the likely impediment, are you aware of the MED safety app that was introduced recently during COVID 19. Do you think it can integrate well with the existing surveillance system or can it be implemented fully as alternative or to complement this manually system? What is your experience with it? What are the challenges with it and what are the advantages are of that and what are the challenges implementing it?

Participant: One, at least let me start with the challenges. I think I was part of the training. Even utilization, the people that are supposed to use the app they found it difficult to configure it even to be able to put the required data element inside and transmit it. Then sometimes some people will complain of network availability across board and it is something that you have to use it all the time if you started using it. But if you are, you know the, when it comes to digitalizing data processing for assessment, I think it will make the system simpler because data is submitted from the primary source directly and you cannot be able to falsify. There will be no room for modification the data can submitted on time and there will be no room for falsification after the data have already been submitted.

Interviewer: That is one of the advantages of using med safety app

Participant: Yes, you cannot be able to alter data after is already been captured or may be after collecting the whole you can be able to and change the figure but when it is in the app immediately you send the data you can be able to have access to it to make any adjustment or correction.

Interviewer: But generally speaking, outside this, what are the other challenges with optimal detection, reporting investigation and documentation and use of data for AEFI. Generally, outside the nuances of the possible use of med safety app as being piloted generally speaking what are the other challenges?

Participant: To me I can, the AEFI what is lacking is feedback most atimes whatever. My experience all the data generated as far as AEFI is concerned for SIAs and routine immunization, after data is submitted hardly you heard of any feedback and the investigation hardly you see a strong investigation team coming to investigate or even to take care of the management in case there is serious AEFI. There is no provision for free management of victims or somebody that has serious AEFI and there is no good referral system also, you find it difficult to refer a patient if the case is reported.

Interviewer: In addition to that, what is the, in terms of alternative reporting system within the community is there any, isn't there a problem?

Participant: There are problems because hardly you see the community especially let's say the care giver hardly you see them coming to report AEFI cases by themselves. The only time they came to may be when they are coming for next schedule, they say last you gave injection this happened hardly you see that and community members are not aware for them to report. So hardly you see a linkage between the community and the service providers.

Interviewer: Is alright, thank you. How do you, what is your perception regarding the functionality of AEFI surveillance and documentation for routine immunization compared to supplementary immunization activities or outbreak response. Can you describe the procedure, the surveillance system and the procedure? What are the differences, which one is more functional. Those are the things I will expect you to elaborate on.

Participant: Honestly, like during the AESI, you found that there are there will be a set of AEFI committees from the state level to the LGA level. There will be provision of logistics. You have all vaccination across state will have an AEFI kits and there is a real-time data transmission on daily basis. Whatever happens as far as AEFI is concerned everybody will be aware and there is real-time feedback, which the reverse is the case during routine immunization. In routine immunization, most atimes you found that all these are not available. The data is only submitted let's say on monthly basis and people don't follow up. Hardly you have feedback or data analysis been shared at this case reported, these are number of cases, these are serious, these are not serious. But in terms of SIAs, you found out that on daily basis, there used to be a kind of feedback on number of cases reported by LGA or number of cases reported by even vaccination posts at lower level, which reverse is the case in routine immunization. Most facilities conducting routine immunization don't have well- structured AEFI surveillance system, because there are no even provisions for AEFI kits hardly you go and see any AEFI kits. Sometimes, even when you meet service providers, if you ask him what can he do, in the event, there is a serious AEFI you out that he/she has knowledge gap on how to manage even serious AEFI.

Interviewer: Ok, but that is much better handled with SIAs because the training is intensive?

Participant: Yes, the training is intensive. The data collection is intensive, there are logistics are provided and even committee members are sensitized during like training, and community sensitization about the campaign you would found out that topics about AEFI are presented to the religious and traditional leaders which reverse is the case in routine immunization.

Interviewer: Thank you very much. Do you think the, how would you describe the AEFI surveillance and documentation at the health facility level in the state?

Participant: Honestly is weak. In most of the places I have worked is weak. Because you found that out the reporting, you found out that reporting is not true presentation of what is actually happening. You found out that few health facilities are reporting compared to the health facilities are supposed to be reporting. And then the data most atimes you see a lot of data quality issue and sometimes data is submitted is late, the documentation forms, incomplete data in terms of errors in filling the form, missing data element that are not well filled. These are the issue with the AEFI surveillance system at the health facility level.

Interviewer: Ok, in terms of reporting system and data transmission to the LGA how would you describe that

Participant: From the health facility level to the LGA actually might not ... system of data transmission which ... by filling the reporting forms and the line list it will be submitted to the LGA level while at the LGA level they have two different sets of data transmission to the State which one IDS003 and DHIS. So after data is enter you found out that are discrepancies between what is obtainable in DHIS and what is also available

Interviewer: Are there different people that enter

Participant: Yes, there are different people it they ... need to harmonize at the end and even the time they receive the report is different. So these are one of the issues.

Interviewer: Thank you very much then lastly or second to the last based on your experience how would you describe the linkage in term, how would you descrbe the linkagw also make reference between LGA level AEFI data and the existing data management platform like IDS003 and DHIS2 also keeping in mind the timeliness and completeness as well. You touched on what am about to ask but if you can shed more light it will be nice. How would you describe the LGA level AEFI data linkage with the exisitng data management plaform such as IDS003 and DHIS2. Also I will like to make reference to timeliness and completeness of data at thise level.

Participant: The linkage ... different level. THe timeliness of submitting the report from DHIS is different from that of IDS data. So found out that before the data submitted to the DHIS focal person will be different from the one submitted with the IDSR focal person. Most atimes the IDS focal person receive the data before the DHIS focal person. So after he entered if they are submitting their report to DHIA focal person they may actually sometimes you used to increase the figures or submitted one different from the one they submitted to the IDS focal person. I mean in this area they need to have a kind of the data harmonization before you can final data entry between the two entry points (DHIS and IDS003)

Interviewer: Is alright, thank you. But even to the state from the LGA level. From the LGA level to the state what is your experience, what can you say about the timeliness and completeness

Participant: Yes, like from the LGA to the state level you found out that the IDS data usually reach the state before the DHIS data because the IDS enter the data, submit the data to ... and it will be enter prior to the DHIS focal person. So, there is no linkage honestly between the two focal persons as far as timeliness and reporting is concerned.

Interviewer: Bassed on your expertise on experience what would you recommend, what are the recommendation you will put in place or you would push for to improve AEFI sureillance and documentation in Kebbi state and Nigeria as whole.

Participant: Number one, I think ownership, the government should take responsiblity appoint responsible focal persons are very very experience in terms of let's say disease surveillance so that they cab be able to manage the issue of AEFI surveillance at the LGA and state level and they should real feedback. Whenever cases are reported those people need to have feedback of what happened and govenment should provide the needed logistic at the health facility like the data tools, like AEFI kits at least for routine immunization all health facility that are conducting routine immunization officially should have an AEFI kits at all time. Then, there should be management drugs in case if there is a serious AEFI and there be an established refereral system. They identify referral centres if there is a serious AEFi where these cases can be referred and regularly the service providers should be retrain on AEFI surveillance, they should be given refresher training on AEFI documentation and reporting. Then, community members shiuld be sensitised about the need, they should be aware that after receiving immunizatiion vaccine submitting expected to follow and it is not actually a problem if it is reported they need to report nearest health facility and they should follow up to have feedback of what has been reported

Interviewer: Okay, thank you. Any other thing

Participant: I think that is all. Thenthe last but not the least, I think the system should be digitized

Interviewer: At what point, at all levels

Participant: At all levels, right from the health facility to the LGA level, at least that should improve data quality, it will improve data transparency it will also ensure accoutability.I thin at all levels.

Interviewer: Thank you very much for the your time and the opportunity to take part in this study, I am very greatful, we shall, I shall adhere to the ethics of good research in terms of your right to privacy, your right to anonymity and the use of data. Thank once again. Bye bye.

Participant: Thank you
