# Supplementary material for: Barriers to optimal AEFI surveillance and documentation in Nigeria: Findings from a qualitative survey
Source: PLOS Glob Public Health. 2023 Sep 8;3(9):e0001658. doi: 10.1371/journal.pgph.0001658 (PMC10490937; doi:10.1371/journal.pgph.0001658)
Supplement: S1 Data — (ZIP) [file pgph.0001658.s002.zip › Transcription- interviews/PHD IDI WITH DIRECTOR OF IMMUNIZATION.docx]

PHD IDI WITH DIRECTOR OF IMMUNIZATION

INTERVIEWER: Are you aware of the CDC surveillance system evaluation attributes?

Participants: I am aware of it and I have heard about such.

Interviewer: would you say that the current AEFI surveillance system in Nigeria is simple, flexible, acceptable, and sensitive enough to inform vaccine safety consideration? Kindly explain with these attributes.

Participant: sometimes, it is really fairly acceptable, and in terms of flexibility, it is also fairly flexible in such a way that; flexible in the sense that as you know we have three levels, national level, state level, LGA level and health operational level -it has to be really flexible across the levels.

Interviewer: do you think it is simple to operate?

Participant: it is fairly simple. Because of the educational status of our health workers at the lower level I cannot say it is simple as it is. But equally at some levels, it is simple

Interviewer : so the simplicity is affected by the educational status of at the operational level, because there is low level of educational operators at the primary health care level, so it is a challenge and becomes challenging for them, making it less simple.

Participants : yes

Interviewer : in your working experience over the years, because you’re the highest person when it comes to routine immunization or all matters of immunization in the state, do you think it is sensitive enough to provide enough data to guide vaccine safety consideration? Where you able to pick all the cases? Do you think that the data being generated is robust enough to guide vaccine immunization consideration.

Participant: it is (sensitive) during the SIA and OBR more than routine immunization, because during RI there is drastic under reporting of AEFI case because of the personnel involved during the activity. So AEFI reporting is low during RI than during SIA.

Interviewer: Would you conclude that the current AEFI surveillance system being operated is not strong enough to inform vaccine safety consideration or how would you describe it? Based on the amount of data, quality of the data based on the sensitivity, and some of these characteristics, how would you describe it capacity.

Participant: the effectiveness is a little bit fair, based on my own opinion or view, it is fair enough to promote vaccine consideration.

Interviewer: do you think the output from the AEFI surveillance system and documentation can inform demand generation based on vaccine safety findings/data?

Participant: really depending on the educational level of our caregivers, really it matters. Also, the lack of proper sensitization concerning AEFI contributes towards low demand generation during our RI activity because demand generation is very sub-optimal for RI than during SIA. Because the level at which our activity is being publicized is always at optimal level for SIA compared to RI services.

Interviewer : do you that the vaccine safety data based on the system/ or the information’s from that data is adequate compared to provide communication or information that can improve demand generation or adequate regarding the safety of vaccine consideration?

Participant : it is fairly adequate .

Interviewer : So you think it is also largely affected by the quality of active health worker we have at that level?

Participant : Yes.

Interviewer : Do you think the current system is sensitive enough to pick the AEFI- is it representative of the true burden of AEFI in our population either from the community or from the health facility?

Participant : yes its fairly sensitive to pick the cases (fairly representative).

Interviewer : what are the challenges impeding AEFI surveillance system based on your working experience in kebbi state? From the point of detection, collection, reporting, to documentation or use of data for action.

Participant: from detection, reporting and the use of data for action, to me, really there are a lot of challenges like the WHO used to locate some strategic places and some health facilities even from the herbal people, traditional bonesetters (community informants) just as their focal points to ascertain AEFI. You know that it is only WHO that is passionate about it, the state government is not helping even financially to improve the reporting, detection, and documentation and use of data for action.

Interviewer: Apart from the challenges with inadequate or lack of financial support to manage or run the AEFI surveillance system, what other operational challenges are limiting optimal data generation, data reporting, data analysis, use of data for actions? What are the problems at the facility level or the community level, or even at the managerial level, have you seen other challenges that needs to be addressed?

Participant: the other thing apart from funding is the mind set of our people (health workers), their level of commitment is sub-optimal and there is a low level of education of our health workers at the lower level, lack of proper data archiving system even in our LGAs. The surveillance peer review showed a lot of data archiving problem

Interviewer: So, what other challenges or bottlenecks from your experience is there?

Participant: Weak supportive supervision and reporting system, at the lower There is also work overload i.e., manpower shortage. For example, only one or two technical personnel running RI, ANC services, all the activities at the health facilities. They are overworked and do not mind reporting AEFI. During the SIA the partners that are funding the SIA really have interest, but in RI the nationals does not show interest in AEFI cases. At the government side, there is no much support. At least funding should be provided to support AEFI surveillance. There was an immunisation held in Kano where it was suggested that an agreed amount should be dedicated to support AEFI activities.

Interviewer : what is your recommendation regarding the quality data given the fact that it is heavily manual, do you think it is also a challenge? If so, do you think we can afford the electronic data management or electronic reporting and data management system.

Participant: looking at our economic resource, and you know that electronic data collection and reporting has its own challenges. You know it is expensive and the level of education of the health workers at the operational level is low, so they can’t really handle those electronic softwares. Sometimes, there is network problem which really affect this software or electronic operating system. For example, when filling the ODK during supervision, sometimes, there are network challenges making it difficult to submit. Non-interest of the government to support electronic data management system for AEFI.

Interviewer: what is your perception regarding functionality of AEFI reporting and documentation compared to that of SIA or outbreak response? And give reasons.

Participant :Only when it comes to the matter of functionality, during SIA more cases of AEFI are reported, than during routine immunization. That is because during RI, not even service provider will take his/her time to explain the six key messages among which AEFI is included, they don’t take their time to explain to the caregivers before or after the immunization, but during SIA or OBR, there are some key messages that they share with the caregivers about the importance of this AEFI reporting.

During routine immunization not even health workers will even remember to tell the caregiver to wait at least for ten to fifteen minutes so that the child will be observed for possible AEFI which may likely occur, you know the mind set and the general attitude of our health workers towards reporting of this AEFI is sub-optimal because they think that if they report AEFI, it is as if they committed a crime/ an offense. But it is not an offense to report AEFI cases, in fact you are helping the system when you report these cases to develop. And secondly, during SIA the teams go house to house and reminding households on the importance of reporting AEFI to the nearest health facility.

Interviewer: is there a difference in the procedure of AEFI surveillance and documentation between RI and SIA or OBR?

Participant : really the procedures are the same, but in SIAs, all the data tools and kits, all the information are given, tally sheet is filled in immediately, but during RI situation many atimes most of the health facilities or health workers have improper documentation of tally sheet or AEFI cases.

Interviewer: how would you describe the AEFI surveillance and documentation at the health facility level in the state?

Participant : to really go straight to the point, it is very very sub-optimal

Interviewer : how would you describe reporting system and data transmission to the LGA?

Participant : the reporting and data management transmission of the LGHA also is very fairly optimal.

Interviewer: in terms of timeliness and completeness, how would you describe it, especially regarding its linkage to DHIS2 and IDSR?

Participant: the level of timeliness and completeness is not that good, it is very sub-optimal because some of the health workers in the LGA are less concerned about transmitting the AEFI reports until the M&E officer remind them to do, then they will just quickly rush through it and it will lead to data entry errors.

Interviewer: based on your expertise and experience, what would you recommend to improve AEFI surveillance and documentation in Kebbi State or in Nigeria as a whole?

Participant: I would recommend that health workers or operators needs to be retrained properly again because there is a saying that if you want to plan for a year you sow a seed, if you want plan for a decade you plant a tree, but if you want to plan for a century you educate the people. If you educate people, they will really do what is required or expected of them to do. The training should be at all levels, from the lowest level to the highest level. Because if it is good at the operational level, then the aim is achieved. Secondly, government also should consider this AEFI reporting system to support it at all level (prioritizing AEFI surveillance and documentation). There should be accountability, who is supposed to do what at each level. The level of commitment towards the goal or to their work and make sure that the right thing is done as it should.

Interviewer: what about those at the community level, what do they need to know/do?

Participant: At the community level, training of informant on reporting AEFI- we need to learn from what WHO did on reporting of AFP reporting, that they trained the community informants and everybody was fully involved.

Interviewer : thank you very much .
