# Supplementary material for: Barriers to optimal AEFI surveillance and documentation in Nigeria: Findings from a qualitative survey
Source: PLOS Glob Public Health. 2023 Sep 8;3(9):e0001658. doi: 10.1371/journal.pgph.0001658 (PMC10490937; doi:10.1371/journal.pgph.0001658)
Supplement: S1 Data — (ZIP) [file pgph.0001658.s002.zip › Transcription- interviews/PHD IDI WITH SDA.docx]

**PHD IDI WITH SDA**

**INTERVIEWER: Are you really aware of the CDC SURVEILLANCE SYSTEM EVALUATION ATTRIBUTE?**

**PARTICIPANT: NO**

**INTERVIEWER: OKAY, you don’t know anything about it, you have not read about it.?**

**PARTICIPANT: Yes, I haven’t**

**INTERVIEWER: Okay great thank you, do you think that the AEFI surveillance system in Nigeria based on your experience in Kebbi state is simple, flexible, acceptable, stable and sensitive enough to inform vaccine safety consideration?**

**Participant: Yes absolutely the AEFI SURVEILLANCE SYSTEM in Nigeria and especially in Kebbi State is simple. What I mean by simple is that it is adaptable, and it’s easy to work with especially at the LGA level and also at the health facility level because from my experience the RI providers are the main actor in AEFI SYSTEM ACTIVITIES and whenever during the training when they were told about the expected task ahead of them, all of them assimilated it well and understood the scope, and if you go to the LGA LEVEL in their health facilities, you will see the level of documentation they have, although there are other things to improve on but their documentation is good and they take corrections immediately and improve on it. So the system is effective and simple.**

**Interviewer: is the system simple, flexible, acceptable, stable and sensitive enough?**

**Participant: yes, it’s also flexible because as you can see, when AEFI surveillance was re-introduced, though the surveillance for AEFI has been there, but not as effective as now. When it was remodified by calling it serious and non-serious, before we were using a terminology of mild and serious but after remodifying it, the people quickly adopt to that and adjust. So that make it obvious that the system is simple and the terminology is not that ambiguous for the health workers to understand. And in the past there was no issue of novel oral polio vaccination exercise, but when they came up with the idea of using the nOPV2 to control the outbreak of vaccine direct polio virus so they build on their system and the existing AEFI surveillance system to ensure that any issue of AEFI is to be reported immediately at the vaccination post. And for those that do not observe that at the vaccination post, they can as well go to the health facilities to tell them their observation after vaccination at the post.**

**By doing this, it shows how flexible the system is and the system is the same method used for covid 19 vaccination. Because during covid-19 vaccine there was issues of AEFI and the system was able to pick the AEFI at the vaccination post. And for some people were unable to wait for that fifteen minutes, so when there were observing this issue they quickly go to the nearby health facilities and they were attended to and those case were documented as well. I can say that it is flexible cos it allows new vaccine introduction or new vaccination exercise to benefit from the existing system**

**Interviewer: thank you. So do you think that its stable in terms of its availability and it capacity to it reliability, will you say that?**

**Participant: I can say it is partially because it involves three things. One, the data generated from the health facility, is the data enough to make informed decision. So when we look at that, we can say it’s not truly stable because the issue is that there are times we have enough data and large number of cases especially from the health facilities and sometime we won’t have enough data.**

**Interviewer: can you make yourself clearer please. When you say data, are you talking about the transmission? Is the system able to collect data adequately, manage it from one level to another without failure?**

**Participant: no, that’s where the issue is.**

**Interviewer: can you elaborate ?**

**Participant: documentation at health facility, that one is adequately done. But in terms of transmission from health facility to LGA level, and from LGA level to state that’s where we have issues. because at health facility they document everything all the AEFI case are reported during that particular month, but from LGA to the state that’s where the problem or issue occurs because at times you have to call on the DSNO that is meant to give you that line list before they give you the line-list and the line-list given to you might be different from what is on the DHIS, so for probing further, that is when they will go back to the facility and collect the authentic/remaining data.**

**That is why I say it’s not stable because, during the transmission process some information are missing**

**Interviewer: the stability, to rate it, will you say it’s not very stable, is it fairly stable or not stable at-all. Based on the explanations you have provided.**

**Participant: I will say it’s fairly stable**

**Interviewer: okay, in terms of its availability to be operational when needed, do you think it’s available?**

**Participant: Yes, that one is available because they give the caregiver a space to stay for some minutes to observe if there will be an AEFI and also they inform the caregiver about the possible side effect of the vaccine and ways to manage the effect, but if they cannot manage it properly, they are given referral center’s close to them.**

**Interviewer: do you think the data management/ data being generated from the AEFI zonal system is of high quality, useful and timely to inform vaccine safety consideration?**

**Participant: the data is partially of high quality in terms of quality because it is not accurate. Then in terms of timeliness it is also partial. And in terms of usefulness, what we do is that immediately we get the data from the LGA level, we quickly analyze the data, and ones the data are being analyzed, then the decision makers usually at the top level will be able to use the data to make an informed decision, especially, when you look at this on-going covid-19 there are issues of vaccine efficiency especially among the health workers .With the aid of the AEFI data generated that was able to resolve this issue and people came out in large number to collect the vaccine, thank you.**

**Interviewer: Alright, thank you very much, thanks for the opportunity.**

**Based on the above discussion, will you say that the current AEFI surveillance system is 1. effective and robust enough to inform vaccine safety consideration? 2. Effective and robust enough to informed vaccine medication strategy or to guide communication strategy? Or to guide communication strategy towards inform demand generation for immunization.**

**Participant : Yeah, the first part of it is partially effective, as you can see from my explanations, in terms of timeliness of data being generated the timeliness is not of high quality and for you to be able to use any ….**

**Interviewer : you mean it is not very timely?**

**Participant : yeah it is not very timely and also, in terms of completeness too, it is poor. Because most of the times we will be the ones to be hammering on the LGA DSNO before we get the complete data from the LGA level. So this alone tells you that there are gaps in terms of data reporting in terms of data management from the LGA to the state level**

**Interviewer: if you were to put it in percentage for the timeliness generally from the experience, for example, the last year data if you are to put it in percentage for timeliness of course we know the benchmark is 80% while for completeness is 90%, so what will you say?? If you are to summarize in proportion for the state.**

**Participant : so in terms of timeliness for last year it is 70% and completeness for last year is 95%**

**Interviewer : for this year?**

**Participant: For this year I will say 90% because we are not there yet, but we are still trying.**

**Interviewer : Do you think it can also , the system the current system can inform the development of communication strategy that can guide the demand generation for immunization ?**

**Participant : yes absolutely, based on my experience and looking at the pool of the data coming from the LGA, it shows all kinds of reasons and these reasons are numerous cutting across all the 10 available reasons and others that are not part of the defined reasons. So this alone tells you where to channel your communication to and also tells you how to educate the mother, so that the issue of AEFI will not be the reasons for them not to be coming to the health facility to get their children vaccinated.**

**Interviewer: that’s very good, thank you very much our participant am very grateful**

**We shall move on to the next question which has to do with the bottlenecks, what are the challenges or bottle-necks that are impeding on AEFI, AEFI SURVEILLANCE documentation i.e., from the point of detection down to investigation, documentation and even the use of data in Nigeria based on your work experience in Kebbi State?**

**Participant : okay good,**

**Interviewer: What are the challenges based on your experience in Kebbi State from the LGA level down to the state and in the national .**

**Participant: Okay. One of it I can call financial support because for the DSNO one of the issue they have been hammering about health facilities in hard-to-reach locations. They may need a kind of financial support to move from the health center to another and collect and collate data to the state level; 2. Security issues, is another major issue especially for those LGA in the southern part, there is a risk of insecurity- bandit attack and so on . 3. It is on the level of documentation because documentation is paramount , it tells you what you are doing good and where you need to improve on, so the level of documentation in some of the health facility is very poor, not all but some selected health facilities especially in the rural areas where they know that they don’t receive visitors on routinely bases, so they tend to be idle in terms of proper documentations of these AEFI cases. They don’t do proper follow up to see the outcome whether a child truly has AEFI or not.**

**And looking at the health facility down to the LGA level like I told you they have to be pressured before you get the data at times, so if there is availability of finance, then the DSNO will be able to move freely collecting the necessary data from health facilities down to the state level and there won’t be any issue of documentation or data transmission issues**

**Interviewer: so if I understand you, you talk about poor supportive supervision, finance challenges that constraints movement of DSNO ‘ you talk about insecurity and poor documentation. Are there any other challenges in the operational level?**

**Okay thank you very much, what about other levels, is that where the problems are limited to? Are there other issues at other levels because AEFI surveillance and documentation does not only have operational at the primary health care facilities?**

**Participant : Yeah, it goes beyond that**

**Interviewer : Or are you just looking at it from data perspective alone?**

**Participants: Yes, I am looking at it from data perspective and if I should also go beyond that, I can also have issue with the care givers because at times this care giver don’t want to report whenever their child is having AEFI so they would not want to go back and report and when you ask them of what prevented them from coming to the health center to tell us what reaction or side effect your child experience some will tell you that they are not mobile and it is only when you have hard to reach team or outreach session that is when you know that these are they challenges and this outreaches are not being conducted on a routinely basis due to financial constraints that is when you tend to identify that a case was not reported .**

**Interviewer : thank you very much once again. What is your perception regarding the functionality of AEFI SURVEILLANCE AND DOCUMENTATION for routine immunization compared to that of supplementary immunization activities or outbreak response/SIAs?**

**Participant: Yes, for the routine immunization AEFI and also the outbreak response in comparing the two, I will say that the outbreak response is effective because there are a lot of interest in that one and a lot of reporting channels, also the interest from the national level because people will be asking about the line list and other necessary documents and there are high level of supportive supervisions, so the health worker cannot in any way not provide proper documentation of AEFI especially during the SIAs. So, all of them will be up and doing and even when they don’t document, and a senior supervisor sees that they are not documenting information, they will be told and cautioned to do so. This supportive supervision makes it better compared to routine immunization and also for the AEFI, a waiting area is provided for the caregivers compared to routine immunization. Also, during SIAs, they tell the mother or care giver of all the necessary information and side effect of the vaccine compared to the mobilization for the routine immunization. In summary, the AEFI surveillance of the SIAs is more effective compared to the routine immunization.**

**Interviewer : thank you very much .**

**Can you describe the difference in AEFI surveillance and documentation procedures the difference between RI and SIA or OBR?**

**Participant: Yeah, one of the differences is the data tools for surveillance and documentation of SIA and AEFI surveillance for RI. In the SIAs, you already have a prior knowledge of the number of AEFI cases reported and you also know the number of line-list your expected for the SIAs while for the routine immunization there is no such prior knowledge. Let me break it down, in the data tools for SIA AEFI, maybe the daily call-in data, there is a room where you collect the numbers of AEFI reported cases by LGA or by vaccination point, but in the routine immunization, you only collect the data at the end of the month and it makes it difficult to keep proper documentation.**

**Interviewer : is it timeliness of the data?**

**Participant : yes, the timeliness of the data and also the supervision of the data, so it makes it difficult for you in the routine immunization even if the data is not complete to know that this data is not yet complete and it is only when you compare what you have on DHI 2 platform and also what you have on the line list that you know that one is more than the other and there is need for harmonization. But for the SIA, it makes it simple, and you know that on this day we have a particular number of AESI from a particular LGA and therefore the line-list is supposed to correspond with each other when documenting. Then, on the part of detection, there is prompt and immediate detection in SIAs, there are no detection or promptness of AEFI on routine immunization and non-compliance is reported immediately in SIA but for RI it takes time before being reported and the factor behind it is the mobilization part of it and also documentation part of it.**

**Interviewer : so in SIA it is there is proper documentation but in RI there are no proper and prompt dictation.**

**Participant : yes**

**Interviewer : do you think the AEFI reporting and documentation at the health facility and LGA level are filled properly into IDHR and DHIS 2? You can provide reasons for your answer.**

**But to guide you, lets approach the question in this manner; how will you describe AEFI surveillance and documentation at the LGA and health facility level in the state? State your reasons for your description**

**Participant :Okay good, the documentation of AEFI at the health facility is perfectly done .**

**Interviewer : surveillance and documentation**

**Participant :the surveillance and documentation are perfectly done based on my experience because the RI provider try as much as they can to ensure that whenever they have issues of AEFI case they document it immediately, but in terms of detection there are some gaps, the AEFI that happens, the event that happens at the health facility is documented immediately , but the events that does not happen at the health facility often time miss because of the fact that the care giver don’t report and they also don’t follow up, basically these are the two things.**

**Interviewer : thank you very much. But do you think that they actually have adequate data tools for documentation?**

**Participant: they do.**

**Interviewer : thank you very much , so how will you describe the reporting system and data transmission to the LGA level?**

**Participant : I can’t say it’s perfectly good,but its not very bad , it fairly good because it’s not very bad**

**Interviewer : can you use an adjective then? A simple adjective to describe it .**

**Participant : its partially perfect, because you can see , thank God the nationals adopt the use of DHIS2 platform and also allows us to line-list the cases of AEFI, so for that we will be able to use only one as a determinant to see if there is a gap or not. So what I mean by that is that the M&E enters the data on DHIS2 platform while the DSNO prepares the line-list from health facility , so where the M&E is having data gaps, the DSNO tells us by the line-list we have and whenever the DSNO is having gap, the M&E data on DHIS2 platform also tells us by stating the numbers of AEFI in each LGA and if it does not tally with the DSNO line list we try to breach the gap. So combining the two together is perfect.**

**Interviewer : how does the reporting system work?**

**Participant : the reporting system from health facility to the LGA is effective because the health facility is the primary source and they document whatever AEFI case they have to the LGA level and the M&E goes to the health facility to take the summary not the line-list and not the details and they enter that summary into the DHIS2 platform on the fifteen of the preceeding- month, while the DSNO also go to the health facility to take the duplicate of the line-list and summit it at the state level and this is entered into at the AEFI access data base, and when you export it goes into the excel to analyze and from there you can know what the issues are and also reasons for each AEFI , so it is well detailed compared to the M$E data.**

**Interviewer : are you saying that there is a different data base for AEFI at the state level? ,I mean there is a different database that can be used to capture the linelist of AEFI? And how does that fit into the IDSR because you only mention that of DHIS?**

**Participant : yes thank you very much, so this particular data base allows you to capture all the information about the patient ,everything about the patient while the IDSR is looking at the number , not the reasons or the place i.e. residential address or person i.e. age group, the end result i.e. is the child alive or not? But for the data base, it captures everything like the vaccine given that led to the AEFI, the age ,the residential address etc and also the end report of the outcome of the patient is she/he dead or recovered? It caputers everything so it more detailed than IDSR and also the information on DHIS2.**

**Interviewer: what is the name of the data base AEFI?**

**Participant : it is called AEFI DATABASE and it access, and its being sent from the national to the state**

**Interviewer : and so the LGADSNO fills it? Or who fills it?**

**Participant : the DSNO have capacity but the state the WHO office aid the state and enter those data.**

**Interviewer : is that the routine practice?**

**Participant : yes that is the routine practice**

**Interviewer : thank you very much, so based on your experience in totality, do you think, how will you describe the linkage between the existing data management platform such as IDSR data and DHIS2 and others, and I will like you to mention the issue of timeliness and completeness of this reporting linkage to all these platform.**

**Participant : thank you very much, already I told you that there are inter-connected to some extent and its allows those at the state level even though not present to know where there are problems and how to harmonize your data and especially on timely manner.**

**For the DHIS2 the deadline for the data capturing is usually on the 15^th^ of the following month, so for example, we are in June, it is expected that by July, all the data is collected on or before 15^th^ of July and for you not to wait till the 15^th^ of July, you get the information on IDSR because it gives an insight of the AEFI cases at LGA level because from the health facility to the LGA level they are expected to submit their data latest 10^th^ and also the DSNO brings that data and line-list to the state level and the dead-line for submission is on the 7^th^ of every month. This shows that data will be readily available and so there is timeliness because line-list is always ready before others and IDSR will be ready before the DHIS2 data is ready. But when your capturing the line-list into the DATABASE you would have all the information needed and even allows you to probe further and check where there are lapses and make necessary corrections. So, the system helps in guiding you to collect proper data harmonization and also gives room for data quality.**

**Generally, the timeliness of the data is not there especially from the LGA to the state and this is due to some of those reasons mentioned previously, but there is timeliness from the health facilities to the state.**

**Interviewer: Thank you very much for this information and we shall confer the findings to the stake-holders and GOD willing we will inform and improve public health in kebbi-state and in Nigeria as a whole .**
