# Supplementary material for: Barriers to optimal AEFI surveillance and documentation in Nigeria: Findings from a qualitative survey
Source: PLOS Glob Public Health. 2023 Sep 8;3(9):e0001658. doi: 10.1371/journal.pgph.0001658 (PMC10490937; doi:10.1371/journal.pgph.0001658)
Supplement: S1 Data — (ZIP) [file pgph.0001658.s002.zip › Transcription- interviews/PHD IDI WITH SUVEILLANCE FP from NPHCDA .docx]

PHD IDI WITH SUVEILLANCE FP @ NPHCDA

INTERVIEWER: Are you aware of the CDC surveillance evaluation attribute? And equally do you think the AEFI surveillance system in Nigeria is simple, flexible, acceptable, and sensitive enough to inform vaccine safety consideration.

PARTICIPANT: Yes. I think it is flexible, simple, and sensitive. Not all that sensitive enough but sensitive enough to detect or pick, confront and investigate cases. The general overview of our AEFI surveillance system i.e how it operates is that cases are being reported by the health workers that are in constant communication with caregivers or mother during immunization. The communities have been sensitised on how to report cases. Most of our cases as you mention earlier mainly are being reported during campaign of SIA as against routine and efforts are being made to encourage reporting during routine immunization. No doubts there are a lot of challenges but compared to other countries, I would say our reporting is sub-optimal and efforts are being put into series of training for our health workers at lower level for reporting cases. As of last month, we were number four in terms of reporting in the African continent, South Africa being the first. Talking about sensitivity i.e., whether our AEFI surveillance system is picking cases as it should that has been handled because series of training are being conducted for the DSNOs those at the state level responsible for reporting AEFI cases when it occurs or in the immediate appearance so that they can report cases immediately especially on this new vaccine called novel oral polio vaccine that is being used to respond to the circulating vaccine ravaging most of the States in the country. So the health workers are to report to surveillance focal persons in all facilities in the event of any AEFI cases and then the surveillance focal person reports to DSNOs who in turn reports to the state. And also at the state level we have SDSNO who reports to the state epidemiologist who in turns reports to national coordinating committee, which comprises of staffs from NAFDAC, NPCHDA & FEDERAL MINISTRY OF HEALTH etc. Then at regular intervals, the serious cases are evaluated, screened and forwarded to the NEC i.e., NATIONAL EXPERTS COMMITEE ON AEFI and their duty is to carry out a causality assessment to see if there is any relationship with the event that has been reported and the vaccine that was administered to the case. Equally, there are some people at NAFDAC that upload these AEFI cases to vigiflow and then to vigibase whereby other people around the world will assess the cases that are reported. Moving on to flexibility, there was a training in 2019 whereby all the surveillance officers/actors from the 36 state were trained in two clusters, southern states were trained in Lagos while the northern surveillance officers were trained in Kano. At that training, previously we were using old reporting forms, three data forms i.e., line-listing form, reporting form and investigation form. Any case that is serious was reported to the NEC members. Currently, there are 14 NEC members who are experts in different fields (Pathologist, Infectious disease expert, pharmacist, vaccinologist and so many others). Let me also mention that the country has a national policy on AEFI surveillance which guides how AEFI surviellance should be conducted in the country and it came into operation in 2018. Prior to this year, the surveillance was not well coordinated but now it is a little bit coordinated whereby at the state level we have AEFI COMMITEES, at LGA level we have AEFI committees and these committees support investigation of serious AEFI and also advocate to the authorities at LGA and State levels for funding and supplies of AEFI kits in the event that we have an AEFI case, these kits will be used to stabilized the case before being referred to a facility where it can be given proper treatment, if it is a serious AEFI cases.

Interviewer : I think you have touch on the structure, and the mode of operation, but I will come back to some of the issues you raised which I will love you to use them as set-back or challenges. For example, the AEFI committees, how functional are these committees? Especially at the lower level, state level and the LGA level, also how functional is our referral system? and at some [point I will ask questions and I could remind you to provide your view on that .

The next part I will ask, in terms of quality of the data of AEFI current surveillance system, the timeliness and usefulness of the data being generated do you think there are of high quality and quite useful to inform vaccine safety consideration, you could use any adjective to exactly describe your view.

Participant : Yeah, I think for now I will say it is of good quality it is improving by the day. We have had challenges before because measures are being put into place to tackle those challenges we face before now. Let me state one example, our case investigation form has four pages but there was a case a serious case that was reported to the national level, the person just scan the first page of the investigation form and then send to us. Now with that page or that documentation she has sent to us there is no way the NEC members on AEFI can sit and carry out causality assessment because vital information are missing which they could use to classify the case. Another case a serious case in the same LGA in Lagos, they sent only a reporting form, but three things must always be submitted to the nationals which are the line-listing (single row), the reporting form (one-page document) and the complete investigation form (four pages) to enable NEC conduct causality assessment. In terms of usage of what we are getting from the field, I will say it not at its best, but we are trying our best to see that everything is brought to perfection, it will take time but hopefully we will reach there.

Interviewer : so you are saying that the quality is not yet there?

Participant : yes but we are trying our best to see it is improve.

Interviewer :Okay great. So overall, would you say that the current AEFI surveillance system is effective and robust enough to inform vaccine safety consideration?

Participant : yes, I will say that it is robust and effective enough because with the tools that we have today, if a vaccine is brought we will speedily accommodate it and the surveillance officers at lower level will send us report. The system has been made so flexible that it adjust to any modification that we will want it to be implemented.

Interviewer : thank you very much sir

Participant : thank you

Interviewer : what are the challenges that may be impeding to optimal AEFI surveillance documentation in Nigeria based on your working experience?

Participant : there are a lot of hinderances or barriers AEFI surveillance in the country which includes; 1. Low detection of AEFI case. There is an indicator that is very important which up till now we don’t know we could overcome the challenge. We have a meeting with the DHIS 2 team today. We have not been integrating the number of vaccinations given with the reported number of AEFI on DHIS platform. But we are working on trying to meet that indicator, so we really don’t know- there is no ratio now for every 100,000vaccination given, there is an expected number of AEFI expected But that is still a challenge to us. We don’t have details of total vaccination given by private medical facilities, even in government facilities you find out that there is inadequate register in most of the facilities whereby these vaccinations are documented. Even the AEFI cases reported from that facilities. In the event of any serious cases are not properly documented. 2. Funding is another major challenge, because the people that will be going out to for investigation or for surveillance will be waiting for logistics from partners especially WHO. So there is no budget line to support AEFI at the State and LGA level. 3 Again, there is insufficient staff at the facility level to mount an effective AEFI surveillance. As we had experience with polio surveillance, whereby there are community informants that complements the work that the regular surveillance officers are doing in the facility or community who report any AFP case to the follow surveillance system we are really hindered by the fact that we don’t have informants among the community because if there are community concern, the community is supposed to report because there are being sensitized, they don’t know that in a campaign mode, 42 days is the maximum time to report any event that is related to the vaccination given to their child or any member of that community. The parents too are not properly sensitized although during campaign mode there is a waiting area after vaccine for 5-10mins to observe and detect any AEFI after immunization.

4. Data tools is another challenge, photocopying of reporting materials is a challenge. But in most cases, WHO is supporting the printing and distribution of data materials. 5. The AEFI committee at State and LGA levels are in most times not functional enough. As such their impact are not being felt by the surveillance system because in the event of any serious case, you find out that it is the family of the victim that takes care of the medication of the patients. Although, we have sent a circular after the training we had in 2019, to all the state commissioners of health in supporting any AEFI victim with medications. Some states have been able to render these services to victims of AEFI cases, but some have really not done much assistance.

6. Feedback although we have started giving feedback to the state on any case reported and that has really discouraged them to send the case to the national level. Because when you send a case to the national level you expect a feedback, but now if the feedback is not complete whether there is causality assessment or none which you in-turn will want to give back to the caregiver of the victim that what happen to that child that they brought has no relationship with the vaccine that was administered to him/her. So that really has affected or are affecting reporting from the community to focal person and then upward to the LGA, state and national. These are some of the factors that are affecting surveillance system in the country. We are doing our best to eliminate these barriers but they still persist.

And there is issue with completeness of the report being sent and some are highlighted in the training we held with a State today. Some spaces ae not being filled in the reporting and investigation form. If it happens that the unfilled space is key to the decision making for causality assessment to be made by NEC. It becomes impossible for NEC to make an assessment. Also if feedbacks are not given, especially when community knows about the AEFI, then if there is no timely feedback, then the programme may be adversely affected (poor uptake), especially for EPI and COVID-19 vaccination and other vaccines that require multiple doses, in the era of social media.

Interviewer: what is your perception regarding the AEFI functional routine immunization compared to SIA and what do you think makes one work better or more efficient than the other.

Participant : We have noticed that reporting during campaign is far more better than during normal routine immunisation.

1. Supervision- during campaigns, supervisors visits the vaccination sites and they do enquire about AEFI recorded or reported, but during routine immunization, not many supervisors visit vaccinations session. The AEFI during routine immunisation are grossly under-reported. 2. Availability of kits and forms: there are forms that every team during campaign vaccination go out with these forms and there are supplies, no stock out of these forms, But during routine immunization, the team hardly go out with these forms and due to that, there are no immediate report of AEFI and verbal reporting of AEFI is not acceptable. During routine immunization, these forms are not always available, and this is one of the difference between SIA and RI.

3. Feedback: During review meetings daily, the supervisors are made to report any AEFI case they had or observed in the area they visited or worked. But in RI, nobody asks you for such reports in the session you have just finished conducting. So you can only report what you want during RI.

Interviewer : how will you describe the AEFI documentation and reporting at the LGA level and how it fits into IDSR and DHIS?

Participant : DHIS is just coming on board, we have had discussions with them so very soon we will have AEFI cases being reported on DHIS platform but for now I will say it is not encouraging but this IDSR this one has not been full handed over to NCDC who are now the custodians of IDSR the integration is ongoing, they have it in the IDSR platform but not much is being reported on that platform .

The integration is ongoing and with time, all AEFI cases will be reported either through campaign or routine immunization from LGA level, state level reports will be on that platform, but for now it is very minimal.

Interviewer : what the role of Medsafety app and how will it work, will it totally replace the current paper system? Are we ready to invest in the requirement to implement the MEC system APP? And is it going to erode the current surveillance system making it totally electronic and if so, how is it going to happen? What are the expectations or is it just going to be at a liberal level or is it fully taking over the space? In other words are we going to abandon the paper work and do we have the capacity to invest fully on this app given the cost and other challenges that may accompany the use of electronic data capturing and transmission system, like the issues of internet network, provision of android phone or tablets? Where are we on this and what is the likely plan, because I know that there are using it for COVID and what has it been like so far? How is it going to plan out with respect to the current surveillance system?

Participant : I think it is a good thing to have the Medsafety app just as we have started with electronic surveillance, before our surveillance was being done manually that the use of paper forms. Now there is a possibility to move from paper form to electronic surveillance, which is a good idea. But Medsafety app is good and it also has its own limitations. What we observe because we had discussion with the promoters of this Medsafety app and we pointed these observations to them so that they can improve the standard of Medsafety app. The observation is that it only covers some aspect of our reporting form and does not take into consideration all the 26 variables in the line-listing form, it does not take into consideration the investigation form, and lab result or any other attachment that will be required to backup the investigation form and linelisting to the NEC members. Now, by the time the say there are going to work on it and improve on it, so as to meet up with our demand because I will say for now that Medsafety app is not useful to us because the information reported are not complete (detailed) and therefore it cannot be used to carry out causality assessment and if this causality assessment cannot be carried out properly using Medsafety APP, then it has not meet up to our needs and that is to say it is not useful to us.

By the time they do what we asked them to do, we shall have a real-time data in a matter of a second from any part of the country to Abuja and the NEC can be quickly assemble our NEC member to make causality assessment. There is no provision for attachment or any other details that can support the causality assessment.

Interviewer : does Medsafety app accommodate for non-serious AEFI ?

Participant : yes it does .

Interviewer : okay so it is not detailed, like you said it doesn’t capture enough information to be able to make causality assessment .

Participant : Exactly, for example, now when you have a lab result there is no where you can enter it on Medsafety app, but if provision can be made for attachment, then whatever you have can be scan and attach and submit it.

Interviewer: Thank you very much my chief. Then lastly, based on your expertise and experience, what will you recommend to improve AEFI surveillance and documentation in Kebbi state and Nigeria in general.

Participant:

1. Adequate funding

2. Proper training.

3. Regular provision of data tools- both paper and electronic

4. Logistics.

Interviewer : what about the issues of awareness? even among health workers and at the community level informant which you mentioned earlier.

Participant : yes, you can add awareness because in training, the focus is on health workers but sensitization is for community informants.

Interviewer : in terms of national no interest/importance has been placed on AEFI surveillance

Participants : Not much, because if they have considered it of priority, there would have been funding and this could be because the people are not reporting of AEFI by health workers, parents or community so the government may feel like everything is okay. If the reporting is good, it will stimulate the government to prioritise AEFI surveillance and documentation.

Interviewer: is there a role of a pharmaceutical company, the vaccine manufacturers to play especially in low-and-medium-income countries for AEFI surveillance? Do they have a role to play?

Participant: Yes, they have a role to play because NAFDAC is engaging them in post-marketing surveillance on all the things that they are selling to follow-up. They are relying on us to provide information or update concerning AEFI cases. They are not doing much to support government to carry out AEFI surveillance.

Interviewer: The NEC meeting, how frequent is it?

Participant : It is quarterly, but when there are cases, we can go beyond quarterly. We can assemble at any time.
