# Supplementary material for: Barriers to optimal AEFI surveillance and documentation in Nigeria: Findings from a qualitative survey
Source: PLOS Glob Public Health. 2023 Sep 8;3(9):e0001658. doi: 10.1371/journal.pgph.0001658 (PMC10490937; doi:10.1371/journal.pgph.0001658)
Supplement: S1 Data — (ZIP) [file pgph.0001658.s002.zip › Transcription- interviews/PHD IDI WITH WHO SURVEILLANCE FOCAL PERSON.docx]

PHD IDI WITH WHO SURVEILLANCE FOCAL PERSON

INTERVIEWER : are you aware of the CDC system evaluation attribute? Have you come across it before or have you read about it before?

Participant : yes, I have heard the word but am not too familiar with it, maybe you can throw more light on it for me.

Interviewer : do you think that the AEFI surveillance system in Nigeria is simple, flexible, acceptable and sensitive enough to inform vaccine safety consideration? However let me give you a little bit of information on the simplicity or what we mean by simplicity and that will guide your response and your insight into the evaluation. when we say simplicity of a public health surveillance system, we are referring to the structure and the ease of operating that system. While ensuring that public health surveillance should be simple, it should be able meet to its objectives and part of the elements include the amount and type of data necessary to establish that the health event is occurring, the amount of data on each cases, the number of organization involved in receiving reports, or operating the system itself, the level of integration with other system, the method of data collection, the time spent on data collection/data management generally, and dissemination as well as staff training and number of staffs required and then the time spent on maintaining the system . Based on this information, would you say that the AEFI surveillance system in Nigeria is simple, and you may want to shed light on your opinion?

Participant: actually the AEFI surveillance system in Nigeria is relatively simple and to some extent there are some challenges especially regarding the reporting and the feedback sharing. For instance, when you look at the AEFI surveillance system in Nigeria in the aspect of reporting, recently there is an introduction of the MedSafety App which is supposed to be an ODK collection for self-reporting. There are challenges with this reporting system in terms of simplicity because for the ODK reporting you need to have an android phone, internet connection and you need to know how to use the ODK for it to be self-reporting. So, the self-reporting is not that simple. Talking of the aggregate reporting from the designated surveillance person from the orthodox or non- orthodox. For example, we have the community informant that report come through for AEFI, it is also not that simple for some of them also, because it is not all of them that still have android phone or know how to use android phone in this ODK reporting but some of them do report. For the orthodox reporting in health facilities, it is simple for them because most of them know how to use this android phone but the challenge for them is usually the workload for this staff because there is limited manpower, only one person that is doing everything. He/She is surveillance officer, routine immunisation officer and clinician. Hence, the aggregate reporting is too much for him or her as he/she might not have time to report as when due. So there has to be a prompting in the system for them to aggregate the report due to some of these challenges. For that of simplicity, it is more at the LGA level and above and reports are being done as at when due.

Interviewer: The use of this MedSafety App, has it been fully operational in Kebbi and in Nigeria especially at the lower level, has it been properly introduced or fully introduced?

Participant: Yes, it has been introduced at the national, State, LGA and health facility levels. However, with the level of dissemination of this module to all levels but the accountability and monitoring and supervision of the use of it is what I cannot say for sure. But based on feedback from the National expert Committee on AEFI surveillance which we did causality assessment of reported AEFI cases, I think there is a very very sub-optimal reporting and the use of this Medsafety app. I think we need to have a refresher training and more of a supportive supervision and monitoring of the use, especially at the lower level.

Interviewer: maybe as well as the tool- android phone, to actually complement it.

What about flexibility i.e., how this currently operating surveillance system can adapt to changing information needs or new operating conditions with little additional information like time, personnel and funds? Can it accommodate those things? Can it be said to be flexible, and can it easily be integrated with other system?

Participant : if this Medsafety app is fully functional, it can be and would be a very good flexible system.

Interviewer : the Medsafety system is newly introduced, do you think it has been properly integrated into the existing conventional AEFI surveillance system?

Participant: From the national level, Medsafety system is now the nationally-recognised reporting system, however at the lower level, there are still reporting through the hard copies and documentation form i.e., the conventional system. However, the integration is where the challenge is, because it has not been fully integrated. In terms of the hard copy or conventional system is still in use, the flexibility is not really there because in the conventional system, for any new vaccine introduction, e.g novel oral polio vaccine (nOPV), AESI surveillance that is being done recently, they have to introduce new data tools for reporting which negates the flexibility of the system. However, if Medsafety system is fully functional, it will be more flexible for the integration of any new vaccine.

Interviewer: in terms of acceptability of the conventional or the current AEFI surveillance system, would you describe it as being acceptable to the sponsors, users and those interested in the data being generated. Would you say that the level of participation and willingness to use it is good enough?

Participant: in the use of the conventional system, those reporting- the users, it is acceptable to them. But for the donors that are interested in the data being generated do not accept this conventional system because data is not flowing from bottom upward. The data seems to stop at a particular reporting channel either at the LGA level or at the state level making the state to have an accumulated and aggregated data for five or six months before pushing it forward, and by the time these data are being analyzed for causality assessment being done, the time has passed for the event to be fully assessed. In terms of acceptability yes, for me the people generating the data but in people that are interested in the data for the use of vaccine improvement and other things, I don’t think it is acceptable.

Interviewer: do you think, or would you say that the AEFI surveillance system in Nigeria currently is sensitive enough to form vaccine safety consideration.

Participant : No, the AEFI surveillance in Nigeria is not sensitive enough to inform vaccine use. For example, the first phase of the COVID-19 vaccination took place for more than two months non-stop. There was a non-stop 40 days, there was another non-stop for 12 days that is up to 60 days vaccination activity and we have LGA that vaccinated more than 20,000 persons, and not a single AEFI case was reported from such LGAs, LGAs like Danko Wasagu- even the largest and most populated LGA then you know that the system is not sensitive enough to report all the AEFI for now.

Interviewer: it is not sensitive enough for vaccine safety consideration?

Participant: yes

Interviewer: do you think the data being generated or would you say that the data being generated from the existing AEFI surveillance system as it is being operated now is of high quality?

Participant : no, because even though we are not getting all the data, sometimes some of the data we get, by the time we analyze it and share feedback, for example during the supplementary immunization activities (SIA) there were report of serious AEFIs from a couple of LGAs because I went there for investigation and we find out that it was data quality error and also we find out that a lot of data that are not being reported is due to the fact that there are so much data quality issues, because some people felt like this is not what there are supposed to report, so the data quality system is not very robust for the present system.

Interviewer: do you think it is useful, the current AEFI surveillance system being operated in Kebbi state and in Nigeria is useful and timely to inform vaccine safety considerations?

Participant : in terms of usefulness, it has to be timely for it to be useful, and like I said earlier on, data reporting stops at a particular point in a reporting channel. For example at the LGA level DSNO or reporting officer might sit on data for a particular data exercise for two to three months and then the state level they also sit on it so by the time the data is being transmitted, it is not useful for vaccine consideration, so I don’t think it is very timely. SO if the data is not time, I don’t think it can be useful.

Interviewer : so overall, if you are to summarize, would you now say that the current AEFI system is effective enough to form vaccine safety consideration?

Participant : No

Interviewer : would you say the current AEFI surveillance system is effective and robust enough to develop communication strategy to inform demand generation for immunization?

Participant : No

Interviewer : do you think it is representative enough, in other words, do you think the current AEFI surveillance system fully describes the occurrence of those event over time and its distribution by population, and by place across the population, place and persons, do you think its robust enough?

Participant : I don’t think so because like I earlier stated, during the recently conducted vaccination, LGA that we have highest persons being vaccinated have a zero report on AEFI, so in terms of place, time or population, it is not fully representative It is when we prompt the LGAs that they start sharing data

Interviewer : stability, do you think the system is stable, meaning that do you think the system is reliable enough to collect, manage, and provide data properly without failure?

Participant : No

Interviewer : do you think the system is available in terms of ability to be operational when its needed, will you say the system is stable? based on these two parameters .

Participant : I think in terms of availability, the system is available, it is a system that can be keyed into if need be, the only thing is that the right materials needs to be used for the system to actively and fully do what the system is meant to do.

Interviewer : so the system is not very reliable?

Participant : yes , but available.

Interviewer : so what are the challenges or bottlenecks impeding AEFI surveillance system in Nigeria based on your working experience in Kebbi and Nigeria as a whole.

Participant : one of the challenges we have is the knowledge of even the person that is having the AEFI to report it to the right personnel (linking the vaccine to the adverse event and then self-reporting) and secondly, at the health facility level, there are no enough human resource because you find out that is only one person that is doing everything and there are work load on that one person, even if the person is willing to report it timely, there are no appropriate data tools for reporting, and up at the higher level, you find out that there are no logistics arrangement for self reporting AEFI cases in terms of free treatment e.t.c. Therefore, people will not be willing to report it (self-reporting) but rather keep it to themselves. Nothing comes as a benefit to them when they report. From the HF to LGA and to State level, there is no logistics arrangement, and in terms of the timeline, it is a bit challenging for the DSNOs to report what they have as at when due when they don’t have data tools at their own level. And at the state level, if they have a signal or cluster for them to go and investigate and then report to the next level, it is a challenge for them because there are no dedicated resources for investigation of AEFI at that level. The only place that we don’t have such challenge is at the national level, where there is a well-coordinated national expert on committee on AEFI with very good, robust secretariat that does the AEFI reporting and collation of reporting from the State level. I think at the lower level to State level, these are some of the challenges we have.

Interviewer: what about at the community level, is there a level of awareness for individuals to even report? What about the functionality of the community informants are they well informed?

Participant : well for the community informants, also in terms of sensitization and awareness of these informants in terms of the tools to report to the next level there is a big challenge because they have to report through a phone-call or probably through a data tool, and these are some of the resources that are not readily available to them, also it cuts across.

Interviewer: what is your perception regarding the functionality of AEFI surveillance documentation for routine immunization, compared to that of supplementary immunization or outbreak response? Is there a difference in the procedure? Is there a difference in the functionality? Can you fully describe the differences?

Participant: there is a vast difference between the two of them. For AEFI surveillance in routine immunisation. For example during routine immunisation session, you hardly find AEFI data tools or AEFI kits, you hardly find the RI provider educating clients about AEFI or even have a waiting period or waiting area to observe for AEFI after vaccination. But for SIA, on the other hand, you find out that these are part of the normal process- there is complete data tools, the activity does not start without the AEFI kits at the HFs, referral center. The referral chain is also well established and being monitored. Throughout the period of the exercise, it is being prompted and ensured to be functional. For senior supervisors going out during SIAs, it is part of the checklist that they use. For routine immunisation supportive supervision, they rarely asked for AEFI, that shows that it is not enshrined in routine immunization activity. The data tools are a must doing SIAs and you see the data on the table, which is not the case for RI. This means AEFI is not seen a core component of RI service delivery

Interviewer: from your experience do relatively causality assessment for serious AEFI cases for the EPI the routine immunization AEFI surveillance compared to SIA or OBR what are your experience in terms of am payable like you said there is a more coordinated more structure when comparing the two what your experience like?

Participant : well, I have not heard any information on causality assessment for AEFI that are being done for AEFI being reported for AEFI RI. Like I said earlier on AEFI for routine immunization are hardly being reported at all in the first place, not to talk of them being aggregated and sent timely, because we you have to share timely data for causality assessment to be done. But for SIA which is well monitored and the national expert committee on AEFI are waiting for the data, which is also well supported with human and financial resources, so the causality assessment is more tailored to the SIA than RI.

Interviewer : in terms of feedback, do you think generally even for SIA and EPI that there is a connection between that national level activity concerning the causality assessment, that the feedback to the lower level exist? Do you think it is operational? If so, then to what level?

Participant : It is not actively or fully operational, am aware of steps that are being recently introduced for data sharing, both feedback and feedforward, and this step can improve the feedback and feedforward. We don’t have a proper feedback for AEFI in a timely fashion

Interviewer : do you think or how would you AEFI reporting and documentation at the health facilities or LGA level fit into IDSR or DHIS 2? However, I would break down the questions so that you will go all out to give adequate or very robust response. How would you describe the reporting system and data transmission from the health facility to the LGA level or State.

Participant: it is sub-optimal because they hardly report a single AEFI case in a sixty-day vaccination exercise. It is very suboptimal.

Interviewer: based on your experience, how would you describe the LGA data system linkage with the existing data management systems, such as IDSR 003, and DHIS 2 and even SORMAS ?

Participant: I think the linkage does not exist, because one, the Medsafety app is not linked with these. And for the old data reporting, I think it is like a parallel reporting system, and that is why we hardly have a feedback or feed forward of what is happening on AEFI reporting and we have to prompt for the AEFI reports. If it is linking the routine reporting system such as RI and IDSR, it would flow in and would not need to be prompted.

Interviewer: in terms of timeliness and completeness, its sub-optimal.

Participant : yes, very sub-optimal

Interviewer: based on your expertise and experience, what would you recommend more than the other to improve AEFI surveillance and documentation in Kebbi and in Nigeria as a whole?

Participant: Firstly, we need to create more awareness to the community and populace and the community informants, health facilities focal persons and how to identify and the reporting channels for AEFI surveillance. Secondly, the Med safety app, which has come to stay, should be supported fully both for self reporting which should be one of the key things in the awareness creation, so that people will know that there is something that they can use to make reports by themselves when they identify that there are suffering from AEFI symptom of a particular vaccine. Also, the Medsafety app, the system needs to improve on their data analysis and give feedback on data that were being reported, in a timely fashion. Through that same reporting channel of that same self reporting, the person should be able to be notified that yes, you are suffering from AEFI of a particular vaccine, and these are the steps to take to manage the situation. Or the person to say no, based on our investigation or findings, you are not suffering from the vaccine. Also, the system should be able to take care of people that felt that they are suffering from AEFI for a particular vaccine for free, in terms of treatment, management and psychotherapy. For example, even medical personnel that took the first dose of COVID-19 vaccine because of some AEFI that they experienced, most of them defaulted for the second dose. There has to be a psychotherapy part of it to reassure them. Also, the national expertise committee need to improve the timeliness of their feedback to the system which will also help to improve the confidence of the community in the system. The Medsafety app will eliminate the use of data. We can provide android phones for cluster of place to focal persons and the community can feed it into it.

Interviewer: but the Medsafety app has its own downside, does it not need internet facility? And what would you recommend for rural areas that have no GSM network?

Participant : In such situation, the data tools has to be made available, so that the focal person that has been assigned there can document case in hard copy then he will now feed it in the nearest point where is an internet facility for reporting to be made.

Interviewer : you talk about work overload for health worker in an health facility, would you like to suggest a way out?

Participant : I think the government should look into providing more human resources in other to fill the gap, like the national suggested. I think that will not just be of help to AEFI only, but also the whole health system needs to bridge human resource gaps.
