# Supplementary material for: Barriers to optimal AEFI surveillance and documentation in Nigeria: Findings from a qualitative survey
Source: PLOS Glob Public Health. 2023 Sep 8;3(9):e0001658. doi: 10.1371/journal.pgph.0001658 (PMC10490937; doi:10.1371/journal.pgph.0001658)
Supplement: S1 Data — (ZIP) [file pgph.0001658.s002.zip › Transcription- interviews/PHD WITH BATCH 1 PROGRAM OFFICERS.docx]

PHD WITH BATCH 1 PROGRAM OFFICERS

INTERVIEWER : Are you aware of the CDC system surveillance evaluation attribute?

PARTICIPANTS : Yes, we are aware of it .

INTERVIEWER : Now going straight to the issue, do you think that the AEFI Surveillance system in Nigeria is simple, flexible, acceptable, and sensitive enough to inform vaccine safety consideration?

Participants : AEFI surveillance is simple, flexible, and it also contributes a lot, in terms of Routine Immunization in our community because whenever there is a program of Routine Immunization in our community if AEFI surveillance is not being done, one will not understand or community may not accept fully the vaccine. Most of the challenges or non-compliance that is gotten in our community is due to poor or lack of proper explanations from our health workers about AEFI. So whenever there is full explanations on AEFI and communities would report cases, and the health worker has reported, if the cases are being assessed without finding out that the problem is associated with the vaccine being given, so the community would now be informed that i.e., the feedback will go back to the community that the problem that has occurred has no relationship with the vaccine given. Despite that there is a little support or medication that is given based on the report so that other community members will not reject the vaccination

Interviewer : Lets hear from other people, do not forget the attribute we want to test or assessed . whether AEFI surveillance is simple, flexible and sensitive enough to detect and then being able to inform vaccine safety .

Participant : Actually the AEFI Surveillance System is flexible, why am saying that is because the system we adopt in our areas, it works because during immunization system, we do give health talk and any case that maybe occurs in the point is always reported to the facility immediately and the LGAs will give us feedback to respective case and it works because we do meet and deliberate during our meetings at the LGA levels.

Interviewer : Any other one?

Participant : I need more clarification on sensitivity

Interviewer : is it capable of picking all potential cases of AEFI whether serious or non-serious? Do you think it is robust enough to pick all the cases?

Participant : ahhhmmm

Interviewer : So Somebody was trying to explain, the question is do you think AEFI surveillance system in Nigeria is simple, flexible acceptable and sensitive enough to inform vaccine safety consideration? You are free to also express your thought about it. Do you agree or think so?

Participant : I think it is partial because based on those things you have listed , based on the attributes, for example, there is no ………..

Interviewer : we haven’t got to that, we haven’t got to the aspect of operational challenges. I want you to concentrate on the attributes. Do you think the system is flexible, do you think the system is easy to work with, Do you think the system is acceptable to stakeholders . eople like you, people like partners, people like sponsors?. Do you think the AEFI Surveillance system is acceptable by all?

Participants: I will say it is acceptable because if there is awareness based on those things you have listed, there is community awareness and health personnel awareness , I think it can be easy .

Interviewer : Do you have any other thing to say?

Participants: I am LIO Aliero LGA, AEFI surveillance system simple and flexible in our LGA because all the staff of the LGA , service providers are well trained to sensitize the community and how to report the AEFI cases, the channel to follow in reporting the AEFI cases, from LGA up to state level and we have a state people that are supervising the programme, partners that are supporting us in developing and generating of this AEFI cases and also during our clinic at the health facility , people are being asked whether there was any adverse event after the immunization to report to the nearest health facilities for action. So with this, the flexibility and the simplicity of AEFI in my LGA is acceptable.

DSNO Sakaba : In fact as my colleagues have said in terms of sensitivity, the knowledge is enough to our staff and also the stake-holders in the LGA level, but the only thing is that not all case can be reported at the LGA, because in my Local Government, Sakaba LGA you will find a facility where only two people are there and they are doing the RI immunization and also the clinic services , so the issue is that not all cases can be captured.

Interviewer : Do you think that the Data being generated from AEFI Surveillance system is of high quality, useful and timely to inform vaccine safety consideration?

Participants : Actually, the data being generated is not of high quality, because, there are a lot of cases that are not reported. In the current surveillance system, only few cases are being reported,some people will report few,…two or three out of thirty because the fear is that where they report this case…………………… which someone should have reported is not good …………………………… as it is expected from them………………………………but some people report it three out of thirty cases …………………………………

Interviewer : its alright thank you

Participants : The quality of our AEFI reporting system is not of high quality just like my colleague has said because most of our staff will think that they are qualified enough to give vaccination .

At the end of the day, they find out that the client being vaccinated come with AEFI, maybe they will think that their work is not all that qualitative maybe that was the cause of that AEFI. And some of the community are not aware of the AEFI because only when community are aware of AEFI that they will report after being vaccinated of their children, that is why I don’t think it is of high quality because only if community are aware that there is or know that this vaccine will have maybe an adverse event to their children either minor or major AEFI because only when you know of something that you can report. So, to me, honestly, I think that there is a gap there, from the health workers, secondly, lack of full knowledge of reporting AEFI by the staff that is why the quality result is low.

Interviewer : Do you think it is timely in terms of the data or the information being generated, Do you think its timely to guide vaccine safety consideration?

Participant: I don’t think it is timely. The AEFI reporting might not be timely, because at the service providing forms, the caregiver was not told to report immediately after being vaccinated at times it is when they come back for the subsequent vaccination, then they will report to the health worker about the AEFI they had.

The quality of what they are reporting are, in fact, in my LGA is not of good quality because it is not timely, let me start with timeliness the staff needs to report it immediately but in my LGA they will accompany it with monthly report, that is when the LGA will be notified, unless if it comes to a serious AEFI. In terms of quality, in my LGA, most of the facilities doing RI, they cover more than ten to fifteen settlements, so the information of these cases of AEFI may not be quality in the sense that not all the community will report on time, at times it will be at the subsequent session when the health worker is asking- has the child felt or experienced any abnormality when he/she was given vaccine before? That is when the mother will start explaining what happen, that is why it is not timely, and it is not of high quality. The DSNO is talking about the case of hard-to-reach, when the service provider conduct outreach service so before they go back for another session the quality of reported cases may not be there because maybe they will go back monthly to that settlement.

Interviewer: thank you very much, based on the above discussion , based on what we have said so far, do you think the current AEFI surveillance system is effective and robust enough to inform vaccine safety consideration? Or how will you describe it?

Participant : The surveillance system in my LGA is weak , the facilities, the health workers, service providers like some have knowledge gap about reporting about AEFI. I could remember that for the past two years they are not reporting at all. Still with the sensitization, training by WHO and other partners to report AEFI surveillance and reporting, we see that those health workers are mobilise or they are more knowledgeable to report AEFI cases in the surveillance system especially last year and this year when they had two trainings about this surveillance system, so we have started seeing their reports in the DHRS and the IDSR and so on, because we have seen a facility where eligible children for immunization will go almost hundred in a month but before you can’t see any DHIS there but now a days you can see that they reported AEFI case , some are investigated, are mostly non-serious AEFI. The health workers before they are in fear of reporting these AEFI cases but now when they see AEFI they are now reporting partially, unlike before .

Interviewer : thank you very much for this, any other person?

Participants : yes sir

Interviewer : go ahead please

Participant : In my LGA I cannot blame staff or the community that because the most important thing is the knowledge of AEFI, most of community and staff knows the type of AEFI whether it is serious or non- serious but the problem is from our staff. At the health facility,we have only one or two personnel , so that is the problem we are having in a particular facility, 2. Health workers is having knowledge on this AEFI, but to report it from the lower level to the higher level will be a little difficult because you may get to the personnel you may find they have a lot of children vaccinated, no how no how , there must be an AEFI but to report the exact figure at the health facility will be a problem and also if you get back to the community also, they have knowledge because they have been sensitized from LGA to the ward level up to the community level where there are told about the types of AEFI and how to report the AEFI and whom to report the AEFI.

Interviewer : Do you think that there is some level of under-reporting? . You are trying to say that there is some level of under- reporting?

Participant : yes

Interviewer : And that may not or suggest that the surveillance system is not quite effective?

Participant : Not hundred percent

Interviewer :Thank you, does anyone has anything else to add ? any other thing?

Participant : In the view of whether we can use the data we generate from AEFI to boost demand consideration..

Interviewer : okay that is the second aspect of the question . Can/will you say that the current AEFI surveillance system is robust enough to inform communication strategy towards demand generation for immunization?

Participant : Yes , the surveillance data of AEFI, that we are generating from health facility in my LGA, to me we cannot use it to boost demand generation because initially there is low quality of data reported in the first place, because most of the facilities are under-reporting, and the community are mostly unaware of when and how and where to report the AEFI to, they are supposed to be told this at the service providing point but that was omitted, so to me the data generated is not enough. What we need to do is to add to the health education of the community and the message that is supposed to be provided at the service point informing the community and the care givers on how to report, what to report and where to report AEFI cases. And also to the community there should be dialogue from time to time about AEFI so that cases will be reported at the health facility. Based on this, so far we are having less cases or not enough cases there are some cases that are being missed, this data that is being generated will not be enough to boost demand consideration because many cases were missing.

Interviewer: thank you very much for your insights into the functionality and the effectiveness of the AEFI surveillance system . Do you think that the cases that we are getting is representative of or related to it sensitivity, do you think that the cases that were getting is really the true burden of AEFI in Kebbi-State? Do you think so

Interviewer : It seems you don’t understand the question, when I say representative am trying to ask you if you think the current AEFI system is representative of the burden of AEFI in the state ?

Participant : As I was saying, the cases reported is not representative because the cases are sub-optimal- not all facilities are conducting routine immunizations, not all facilities are reporting and not all cases are captured in documentation.

Interviewer : what are the challenges or the bottlenecks impeding optimal AEFI surveillance and documentation in Nigeria based on your working experience in Kebbi-State? From the point of detection, reporting, investigation, documentation and the use of the data itself and even feedback?

Participants : No.1 there is no drugs for AEFI cases - serious and non- serious AEFI. No supportive drugs that is given to the community or client over the cases reported. And in terms of referrals, no support of transportation that was given to the health personnel or the caregivers for at least the referral system. Also the feedback, it is not easy to get feedback very quickly to tell the caregivers so that they will feel satisfied with the immunization services these are the few things I would like to say.

Initially, I want to start at the health facility level, most at times when they bring their monthly reports if you ask them about the AEFI report, they will tell you they don’t have data tools to report the cases and sometimes when you go for supervision, when you meet with some health workers when you reach certain level with them, you come to understand that they are afraid of reporting AEFI thinking that they will be punished or be underrated that they are not qualified or patient enough to give vaccinations. So it is one of the barriers to sending or reporting AEFI. Also, AEFI treatment is supposed to be free, most of the time when there is any AEFI case if the caregiver brings the child to the facility, she would end up buying drugs because there is no facility that is giving or treating minor or serious AEFI at our PHCs even at general hospitals, if there is need of admission they will still buy drugs for the treatment .so I think this is part of the challenges we have.

In addition, one of the most common challenges is poor feedback that is to the community as a result of the reported AEFI case and most of the times, they will report the cases late. Sometimes distance from the community to the health facility is too far, sometimes you find out that there is only one facility. The bad terrain in some of our communities, mode of transportation, and poverty make it difficult for the caregivers to return to the health facility to give report of AEFI Another challenge is insecurity , the challenges in reporting AEFI is I think that health workers that fear in them should be killed. Secondly, the community some of them will bring those that have no knowledge about AEFI and they will only vaccinate without giving sensitization on AEFI and the feedback is very poor because they will think it is not all that important to report AEFI to the health facility. None of the HF has AEFI kits, this part of the challenge. We have challenge based on the health workers, poor motivation. Because of the workload in the facilities and there are just one or two health workers conducting different activities- surveillance, routine immunisation and clinical services. Also, there is data quality problem, most of the reports that comes to us you might find out that some data elements are missing and they were not inputted into the right place like age, date of immunization, date of onset, the type of AEFI or second name of the client so all these are the challenges we are facing, ones for the community and ones for the health workers. Care givers enlightenment, at times you will see during the supportive supervision, you will meet the caregiver who brings her child for immunization and ask whether she was told anything about the AEFI she will tell you she don’t know anything about it, so there is need for service providers to sensitize care-givers during supportive immunization.

Interviewer : what is your perception regarding functionality AEFI surveillance and documentation for Routine Immunization compared to supplementary Immunization Activity or OBR ? what are the differences? Do you think one is functioning more than the other? And why is it functioning better?

Participant : what I understand in terms of RI and SIA is that SIA is more functioning because there are some support that comes from the partners organization, but in terms of RI no support. Also, in terms of SIA many personnel are involved, while in RI only those that are assigned to that facility to do the RI are the ones that can do it only.

Interviewer : that means AEFI for SIA is more functional than that of RI, based on those reasons you gave. Any other opinion?

Participant : I am supporting the response from the DSNO, because whenever there is any SIA or OBR you will see the state send some kits to the LGA, even the general hospital, they use to send some AEFI kit to them, but in terms of RI that is not taking place. In SIA, there is a standard given for everybody to abide by. Stipends/ financial support are available during SIAs but in RI there are no standards and stipends.

Interviewer : so in terms of procedures, is there any difference in the way AEFI surveillance and documentation are being conducted for SIA and that of RI?

Participants : It is the same process , the procedures are the same the only difference is giving that incentive during SIA. There is difference, during the OBR there is feedback quickly on the performance of every local government while in RI that is not taken place. Also in terms of SIA, there are some pre-implementation activities that will take place which includes sensitization of community, trainings and all that is what makes the AEFI SIA data more qualitative and more reliable than that of RI.

Interviewer : Do you think AEFI reporting at the facility and or LGA level effectively filled into IDSR and DHIS 2?

Participant : In fact, in my LGA, there are quality issues because sometimes the data that goes into HMIS differs from the one that goes into DHIS2 , also the line-list, maybe they will put in their IDSR that they have ten cases and in the line-list they hardly have five cases, so there is data quality issues and variations in data reporting . Truly, there is discrepancy the data in the DHIS will be different from that of IDSR , because we don’t really know what is reported in those forms.

Interviewer : what is the level of completeness and timeliness of these data flow?

Participants : completeness has already been discussed because not all cases were captured , and then the timeliness is delayed .

Interviewer : so who enters the data into DHIS ?

Participant : the M&E .

Interviewer : how would you describe the data flow?

Participant : it is sup-optimal. it is partially done, because there is communication gap, data entry error.

Interviewer : How would you describe the reporting system and data transmission to the LGA from the health facility ?

Participant : that one from the facility, they document the report in the MHIS summary form and send to the LGA ones it is sent to the LGA, the M&E will now transmit it to the DHIS platform.

Interviewer : so the data from the health facility ie the MHIS goes to the office of the M&E and he enters into the DHIS platform, so who enters the IDSR?

Participants : the data is generated from the health facility through IDSR and also AEFI reporting forms directly to DSNOs and the summary of IDSR is forwarded to the state manually.

Interviewer : so all this processes are they electronic or manual from the health facility down to the LGA and down to the state?

Participant : the AEFI and IDSR is manual

DHIS from the facility is manual, from the LGA to the State is electronic.

Interviewer: thank you very much, do you think that the data of AEFI LGA level, does it linked to IDSR and DHIS 2 well?

Participant : the data for IDSR and DHIS in my LGA SAKABA we use to do data harmonization, we use to compare the data before we forward it to the state. The E-copy or soft copy will be sent through the DHIS and the hard copy will be sent to the state .

Interviewer : in terms of timeliness and completeness, how do you describe the data reporting and transmission ?

Participants : in-fact the data collect from the LGA is not timely., and its not complete because most of the cases were missing from the Health facility, and then data element were not put in place , like data quality etc.

Interviewer : based on your expertise and experience, what would you recommend to improve AEFI surveillance and documentation in kebbi-state as well as Nigeria as a whole?

Participant : I recommend that all the data tools should be in place like line-list, reporting form, investigation etc.

2. Financial Support.

3. Proper sensitization to community and health workers. 4. Someone that will validate data collection 5. I will also suggest that data tools should be one only, instead of separating them into different forms

6. Service provider should be given more training and health workers should be motivated.

7. Government should also see into routine immunization to make it a success by supporting with kits and stipends.
