# Supplementary material for: Barriers to optimal AEFI surveillance and documentation in Nigeria: Findings from a qualitative survey
Source: PLOS Glob Public Health. 2023 Sep 8;3(9):e0001658. doi: 10.1371/journal.pgph.0001658 (PMC10490937; doi:10.1371/journal.pgph.0001658)
Supplement: S1 Data — (ZIP) [file pgph.0001658.s002.zip › Transcription- interviews/PHD WITH LGA PROGRAMME OFFICER BATCH B.docx]

PHD WITH LGA PROGRAMME OFFICER BATCH B

INTERVIEWER: Do you think the current AEFI surveillance system in Nigeria particularly as it is being operated using Kebbi experience do you think it is simple to use? Do you think it is flexible, can be adapted to easily, if you introduce the vaccine do you think it can fit into, the current AEFI surveillance system, do you think if you want to update information on case, do you think it allows it? and then, do you think it is acceptable to all stakeholders? And do you think it is sensitive enough to pick all the potentials AEFI cases?

PARTICIPANT: it is a, it will help to improve vaccine consideration if there is data harmonization from the health facility to the LGA.

Interviewer: I think we need to understand what am trying to find out. please go ahead

Participant : I want to talk about the flexibility of this reporting system in the area of AEFI, when we talk about flexibility now we are doing manual operation of all our health facility which has make it very difficult compared to electronic for instance if you want to make a change , it is not easy to use the manual to make changes because with manual there is no way to update data but in electronic it is easy to make updates of anything that come newly to the system .

Interviewer: Do we have another person that wants to talk about another attribute?

Participants: The system is very simple to understand by our health workers, you can easily understand the case-definition. Talking about the sensitivity of AEFI surveillance in my LGA we are under- reporting any AEFI case because only surveillance focal person are recording the AEFI other health workers in the health facility does not care about AEFI reporting and also the community are not aware of the AEFI surveillance that’s why we are under-reporting the AEFI cases. I want to add more on what my colleague has said, in terms of lets talk about AEFI reporting, I realized that only few health facility are conducting AEFI like in my LGA, we have about fifty six health facilities but only twenty archive and report AEFI, and only the facility that has the training and knowledge of AEFI can report cases that is why there is under-reporting of AEFI cases. Let’s talk about during IPDs, during IPD we have many teams that can go round and search for cases and can cover a lot of areas and with the tools/kits necessary , but during RI we cannot cover the whole areas only areas that are within can be covered.

In addition to that, this sensitivity of this AEFI what I can say here is that the most of our health workers, it is only few of them that know confidently about filling the forms. It is only the RI providers that are invited in training while the rest of the healthworkers are not trained and not knowledgeable about AEFI surveillance and documentation. At least all the health workers should know how to fill the form/report properly so that report can be submitted at least quarterly or monthly, and also our community are to be inform to bring their child when something happens after immunization they use to bring it, but most of the people they don’t have this information that even if it has happen they wont know they are supposed to bring the child to the health centre, it is only when they report it that you can fill the AEFI form maybe a forum should be created in the community so that they can make awareness for the community about AEFI even if when there is no campaign or immunization, whatever happens they will know where to report to .

Interviewer : whether the data being generated from the AEFI surveillance currently being run in kebbi state and Nigeria is of high quality, is it timely, and can it inform vaccine safety consideration?

Participant : there are many differences especially from this AEFI, you will see a health facility immunizing more than hundred persons/ client for RI but report only two AEFI. And after that when you check the linelist sent to the DSNO, you will see more than ten children that shows they had AEFI. For me I can say its partially good data, because there are few reported cases and most of the staffs have a negligence of filling this form, maybe they will submit DHIS 2 and routine immunization data without submitting of AEFI data. The problem of the frequency of data is that some RI provider reporting to the DHIS the DSNO did not enter their data enter their DHIS summary form that is the first issue. In addition to what my colleague has just said, the problem with the data reporting is too much workload and secondly you will see attitude of health workers, and they will not be able to say or release all the information’s to the health workers.

Interviewer : do you think with all these that have been said, do you think the current AEFI system that we are operating is effective and strong enough to inform vaccine safety consideration?

Participant : the system can work partially because most of the time , most of the reports that we use to send do not have feedback, without feedback you will not have confident to continue reports ,that is why the reporting is low because you don’t know the information about the one we have sent, and it will make us just relaxed even if our neighbours or elders ask us to bring report, we will just feel that what is the use of reporting when there is no feedback, therefore this data needs to be given feedback so that it can be improved, because it is partially.

Interviewer : in terms of the capacity of the AEFI surveillance system to guide vaccine safety , the information that you are getting is it enough to guide vaccine safety ? is it enough to inform demand generation for RI or Immunization generally ?

Participant : it will not be enough to guide the safety of the vaccines the reason is because this feedbacks is very important. For me I can say it is partially, to guide vaccine safety because there is communication gap, we cannot give or disseminate the information about the safety of this vaccine to the people around the community especially people that are living far-away from the community so they don’t have information we cannot send information across to them that the vaccine is safe. In addition I can say its complete why because it indicates the data quality , timeliness and completeness of the data, secondly the current AEFI is partially effective because, one there is no feedback from the care giver or health worker when they report serious AEFI case to the LGF, the state team comes and investigate and still no feedback, and also there is under-reporting of AEFI cases and the health workers are not motivated, no support supervision during the RI session and also the work load of the health care workers .

Interviewer: Who else has something to add in terms of effectiveness to guide vaccine safety consideration strategy for demand generation? Or do you all agree that it is partially effective?

Participants : Yes

Interviewer : What are the challenges impeding AEFI surveillance in kebbi state and in Nigeria based on your working experience?

Participants : the challenges includes:

1. Lack or negligence of ICT, no key person to care-giver 2. Lack of enough advice to the caregiver from the health workers to bring their child to the centre if he/she has any effect/signs after immunization. 3. Lack of emergency drugs to manage the AEFI especially the serious cases.

4. Communication gap between the service provider and the care givers.

5.Lack of qualitative supervision especially on that AEFI 6. Lack of enough data tools to report those cases or Mismanagement of those data at the health facility

7. There is knowledge gap to some of health workers because only few have an in-depth knowledge of what AEFI is and how to report it and the appropriate channels.

8. Weak supporting supervision from the LGA team especially to go round and be monitoring immunization process and at the health facility

9. Attitude of health workers towards care-givers. 10. Multiple of data tools and only one person or RI provider is required to fill or handle all these data tools. 11 . Fear of health workers to reporting AEFI think that they will be blamed for the adverse event 12. The form is not legible i.e., hard to read and understand 13. Most are of the teams are LGA teams posted to work at the wards.

Interviewer: what is your perception on the functionality of AEFI surveillance and documentation for RI and SIA or OBR?

Participants : The functionality of AEFI surveillance and documentation is that it dictate the number of RI

Interviewer : What is the difference or why do you think which is more functional? And why is it more functional than the other?

Participants : comparing of RI and SIA , first the SIA is more effective, the reason is because there is more teams allocated to go round and search for cases while in RI, only few facility that conduct this AEFI and report cases. SIA is more effective because there are tracking the AESI line-list deliberately on daily basis while in RI only by the end of the month that reports are done. AEFI surveillance in SIA is more effective than RI because they have supervision, there is effective supervision during SIA and there is AEFI kits and they will treat major AEFI case before referral. There is motivation, they give them something to encourage them do their work and also everyday they will line-list their AEFI case during the SIA while RI is only at the end of the month. Another thing is that there is a form given to IPC to go out and fill and they use to report it daily .

In term of supervision of SIA , both agencies state government and the LGA all the supervisors must specifically stress on this AEFI and even the ODK that are filled during the exercise , it has the provision of AEFI information, whereas in-case the case of RI there is no provision of checking or monitoring what is being done either by ODK or supervisors . During SIA and OBR every team member wants to report the AEFI while during RI all the health facility staff leave the reporting to only service provider for report. Another problem is that right from the state government, there is no person or personnel in-charge of this AEFI, but there should be a focal-person at the LGA level and at the state level to monitor/supervising this RI AEFI documentation and surveillance. SIA is more reporting because they are usually team-up for the exercise.

Interviewer: the next question has to do with AEFI reporting and documentation at the facility level and at the LGA level, how effectively it feed into IDSR 003 and DHIS 2? I would like to have your opinion about the data reporting and documentation and how it is being transmitted form one level to the other?

Participants : the data generated from our facilities to the LGA level is not a real data , it has a lot of discrepancies , for one there was under reporting right from the health facility and this issue of data validation create out a …….. which you will be able to consider the time that DSNO reporting our data will not tell you what M&E are doing ,the data cannot be the same that of DSNO and M&E .

I want to add to what my colleague has said, for we the M&E we have a certain reporting stage for the state up to the last round because our reporting is from 0-14 days of the preceding month while in IDRS 003 we don’t know what time they use to report their data, secondly, we have a copy of IDSR 003 in our DHIS2 data sometimes the health facility in charge of the data that summary they cannot fill it, so they is always a difference in what the DSNO report and what the M&E reports.

Interviewer : Can I ask a question in this linkage? I think from what you have said, there are issues between timeliness and completeness as well .

Participants : yes

Interviewer : And data harmonization is that the case?

Participants : Yes. Between IDSR 003 and DHIS 2

Interviewer : Based on your expertise and experience, what would you recommend to improve AEFI surveillance and documentation in Nigeria?

Participants : I want to talk about data harmonization, like I said earlier we have a monthly data meeting, so it only involve RI provider and DSNO and M&E in-terms of data validation, so for me, I think it is good to invite everyone in that is involve in the LGA to make the data harmonization before sending it to the state. I want RI provider to report what he/she have put in the line-list in the facility to surveillance focal person because RI provider will record in his line-list and at the end of the month, surveillance focal person will update his IDSR 003 without contacting the RI provider, and facility in charge will update and give the summary in DHIS 2 without contacting the service provider with these data are not harmonize, so there should be improve data harmonization. I want to add more on data harmonization, that the in-charge of health facility for RI and the in-charge of LGA data should make sure they check their data again from line-list to have harmony before submission of data.

I want to talk about knowledge gap among the staff, it will be very good if the state government should organize a training or meeting maybe annually or quarterly for all health personnel to build them up. During the SIA all AEFI kits are provided, so I think that all facilities should have or be provided with kits during their normal RI, not only during OBR or SIA. In addition, in terms of workloads, there should increase number of staffs or health workers in the facility. There must be constant Feedback to the LGA or health facility especially in serious case, and there should be someone who is heading the AEFI surveillance.
