# Supplementary material for: Barriers to optimal AEFI surveillance and documentation in Nigeria: Findings from a qualitative survey
Source: PLOS Glob Public Health. 2023 Sep 8;3(9):e0001658. doi: 10.1371/journal.pgph.0001658 (PMC10490937; doi:10.1371/journal.pgph.0001658)
Supplement: S1 Data — (ZIP) [file pgph.0001658.s002.zip › Transcription- interviews/UNICEF.docx]

Interviewer: I will therefore require your consent to proceed with this qualitative interview

Participant: I am very glad to be part and parcel of the interview.

Interviewer: Thank you very much for taking part in this research study and I am very grateful once again. I would like to ask a few questions. The first is: do you think AEFI surveillance in Nigeria as it is being currently operated is simple, acceptable, and sensitive enough to inform vaccine safety consideration?

Participant: Yeah,

Interviewer: You can explain one by one, you may not put the three together

Participant: The system based on documentation is good simple and precise but for its functionality that is where the problem is

Interviewer: So how would you describe it- the functionality?

Participant: Regarding the functionality, there is no willingness on the part of the health workers there are so many lapses. One issue of documentation, they don't take it seriously and if the one that is being documented is not documented properly because you need to know the name of the child, the place, the where the child lives, the address whether you may be able to attract and should be able to know the vaccine that cause this AEFI to the child or sometimes they mistaken everything

Interviewer: Do you think the AEFI system is strong enough to pick or is it sensitive enough to pick all the AEFI cases that will guide us in coming into conclusion about the vaccine safety. Do you think is sensitive enough?

Participant: Somehow it is sensitive enough somehow it is not. You see the AEFI system is good if you look at the WHO trend of AEFI investigation you know it is very very good and very very sensitive to make and forge ahead. But the problem is people who are supposed to conduct this particular AEFI to see how severe it is either minor or major that is where the problem lies at the health facility system

Interviewer: Thank you very much. So you have talked about the ... in a way you have answered part of the next question which has to do with the quality of data from the AEFI surveillance system and timeliness and usefulness of these data. How will you describe it may be once again?

Participant: The quality of the data is too too poor. Too poor and very very low in terms of reporting system until when you go to the field and the community level and the aspect of community engagement. Let's give example in those from the VCM settlements specifically, because a VCM has up to 350 households. A VCM might have up to 100 under one from zero to nine months you will see a child may be among the zero to nine months maybe she has about 100 clients, maybe you have about 30, i.e., those with adverse event following immunization but when you go to the health facility you hardly even see up to five. So, when you ask the results they we tell you there is no paper for documentation, no form, no that and the rest of things to document it. So, these are the things.

Interviewer: Thank you very much

Participant: See, before you continue, even I particularly, my own child was given Penta vaccine and it has such kind of whereby there was abscess and the resting. The mode of the treatment was too too poor, there was no any drug kept seriously for AEFI.

Interviewer: Thank you very much. Thank you. Overall, will you say that the current AEFI system has it being operated is effective and robust enough to guide vaccine safety consideration?

Participant: In paperwork, it is good. But in physical reality it is not. If the system is not working, we cannot say is effective.

Interviewer: Do you think the data being generated from that system can guide communication strategies that will inform demand generation for immunization

Participant: It cannot guide communication strategies because what we are seeing in the society if you go for community survey is higher than what is reported in the facility once you use what inside the facility we will never go anywhere in terms of mobilization.

**Interviewer:** Thank you very much. The next question is what are the challenges or bottlenecks that are impeding optimal AEFI surveillance and documentation from the point of detection, reporting, investigation, documentation and even the use of data for action in Nigeria, based on your working experience in Kebbi State

Participant: I get to found out that societies (communities) are not the problem. Our problems are our counterparts in the PHC level. There is no commitment- RI providers

Participant: On the part of the RI providers, there is no commitment. And the one you see working as RI provider is working on volunteer bases. You may see an RI provider that is giving the necessary vaccine at the same time documenting at the same time (everything at the same time) and yet working as a volunteer. So now a person that is a volunteer can never give is best and those that are being recruited with the full salary are not functional in the PHC that are supposed to be given priorities because you may likely see. You see rationalization of workforce is one of the biggest problems. You will see in a facility that is in the urban settlement you see a lot of personnel up to 20 inside the health facility and no one is even responsible for the conduct of even RI. And these are personnel that are expected to provide interventions and it is only one person who does the job but working as a volunteer. This is one aspect of it. Another aspect of it, you cannot get the AEFI correctly once a report is being made and you (RI provider or health worker) did not document. He/she tells you that there is no material on the ground for documentation, like phones and line list. So, if this line list form is not there or the booklet for line list is not there, so how do you trace somebody. You cannot even trace somebody down to the community level. Even when an AEFI is documented on linelist but follow up is not there to assess the extent – whether it is either severe or minor. Therefore, people (parents/caregivers) get tired so the next vaccine to be received they abscond, they default. So, defaulting again brings another setback for us in terms of routine immunization because it is difficult for you to bring people again from the society down to the health facility. Something you just need to do one or two things to bring somebody back you need to do 20 times of it before the person will be back. So now all the partners are doing their best, but the government need to claim the ownership and they need to forge ahead and look at the aspect of commitment of health workers (RI providers). Sometimes, money or do I say salary is not an incentive. Why I say salary is not an incentive is that you need to a provision for those who are given this service to make them comfortable. One you need to look at the aspect of distance they are coming from. Some will be coming from another LGA to another LGA to give support as a volunteer or as a real staff. Before he comes to that LGA he has been overwhelmed and there is no support give to him in terms of monitoring grant. Sometimes, they will tell you ok there is this for you and the rest. When asking (during supervision) when you go for interview, they will tell you they have not been given this (allowance) for the past 6 or 4 months. So that thing that will make him to go for either fixed post or for outreach services is not there. So, there are so many things ...

**Interviewer:** Any other challenge that is also impeding? Do you think community awareness is good enough for AEFI detection and reporting? Do you think communities are well aware to report AEFI or to notify AEFI, are there informants, unlike the polio informants, do we have informants in the communities that can willingly help us search for case of AEFI?

**Participant:** In reality, we have some community informants. Like some of the Mai Angwan (community leaders) know about it. Some of the community leaders are aware, but not like the time we have the SIA or any outbreak response whereby there are community informant engagements for that. But for this one, it is only some of the stakeholders, like village head, that are aware of this particular AEFI (not all are aware). Some are aware while some are not aware but the larger part of the community informants is not aware so we need much more to do in terms of creating awareness, sensitizing them, bringing them and giving them the right thing to do at the first time when it occur. So, this at least we need to do our best. UNICEF, WHO are doing their best, but the State Primary healthcare Development Agency needs to be much more proactive, to claim the ownership because they are who can be able to call any stakeholder to train and re-train.

**Participant:** Either to train and retrain and to give them the necessary guideline. We are only there to give technical support and sometimes we even forge ahead to the community to provide service. we have limitation, budget e.t.c. The bulk goes to the government.

**Interviewer:** Thank you very much, anything else or any other factor that you think could be contributing to the poor AEFI surveillance and documentation, any other apart from issues about you have summarized, you have talked about the poor ownership by the State Primary Healthcare, you have talked about lack of accountability both financial and otherwise, work overload, poor rationalization of health workforce, especially putting fewer people in the area where they are much more needed and then you talked about data tools. Any other?

**Participant:** Yes, I feel the traditional setting (community leaders), if being brought into this system may be very helpful.

**Interviewer:** Thank you. Then the other aspect, what is your perception, why is it that or what do you think about the functionality of AEFI surveillance and documentation for routine immunization compared to that of SIA or outbreak response. What is the difference, why is that one is more functional than the others, and which one is more functional than the other?

**Participant:** The SIAs is more functional than the others because in SIAs you have so many interventions. Interventions are from partner, WHO, UNICEF, CDC, Rotary, and the rest, IHP, the State breakthrough Action and the rest. You see there are a lot of interventions and there are so many follow-ups, strong accountability framework, so many agencies monitor the activities of one another. Not like routine immunization, only one agency can come without any other agency knowing and understand what is being done. But for SIAs, the EOC is there where all the partners activities are report, queries made e.t.c. But this is not the case for routine immunization.

**Interviewer:** What about SERICC because you mention EOC. There is also SERICC. Why is it that we are not getting that level of scrutiny?

**Participant:** Even in Kebbi State, the SERICC Chairman is supposed to be part and parcel of EOC. I am not even seeing him in the EOC so that we can be able to raise a concern regarding primary healthcare services especially routine immunization. So, the coordination is low.

**Participant:** The SERICC is there. Coordination to make it functional remains a problem. Now as co-partner, I reported, may be, in a particular facility they impose charges imposed on birth registration. I expected an action to be taken by the SERICC Lead or Agency Chairman. Rather, it went up politically and nothing happened. Definitely, mobilization of clients will be channelled towards a distant PHC that is not charging such money for birth registration. Also, recommendations being given after supervision at health facilities in the immunization and surveillance registers are not taken by SERICC for action (none has been taken) or even for a specific facility for proper analysis. Starting from 2000, these are being recommended. It has been recurring everybody has been no action taken. So, once a system is not taking action and is not proactive, the system is not working, to me. And the issue of documentation, the reporting system, the DHIS and the SIAs reporting system there is conflict, the result is parallel to each other- it is not harmonious.

**Interviewer:** Is alright. Thank you very much. So, overall the functionality of AEFI surveillance and documentation for routine immunization is poorer, less effective than SIAs?

**Participant:** Yes

Interviewer: So, what do you think about the documentation at the facility?

Participant: And in the SIAs house to house, you monitor things. But for routine immunization, it is only facility based.

Interviewer: Good, that is another challenge with ... So, the coverage is limited in terms of the likelihood of getting AEFI data or getting the report is limited?

Participant: Yes

Interviewer: Thank you, do you think AEFI document and reporting at the facility and LGA effectively feed in to DHIS and IDSR003. Do you think the reporting is effective in terms of reporting and transmission of data DHIS platform and IDSR003?

Participant: That is why I am saying the data management system is still low. The reporting system is low compared with what is obtainable in the society, with what is obtainable with what people brought and complain in the health facility. So, you see one of the biggest challenges for AEFI, let me be frank to you, is that we have not given the health worker orientation telling them that reporting AEFI is not an impediment to their own system because they think that once you report AEFI that you are not professional enough. Once we remove that stigma from them, a lot of report will be gotten.

Interviewer: The fear will be removed

Participant: Yes, because they may think that once I report two or three AEFI cases, they will say I don't know how to give injections, and this is technical error from me and this is not without them knowing that even the vaccine can cause AEFI, without them knowing there is personnel error. For instance, supposed they give intradermal and went ahead to give through another route. You see this, you need to realize some of thing may not be their faults. But reporting- once you report they will say you did not go to school of nursing, midwifery, you are not a community extension, you are not qualified to give injection here and there.

Interviewer: Thank you

Participant: Also, there are some RI providers according to certificate, they are environmental health workers, and they are licensed to give injection but because of inadequate or no manpower, they are being converted and being trained to give injection. Now, they are In-charges of the health facilities.

Interviewer: I understand, that is fine. Thank you. Based on your expertise and experience, what will be your recommendation to improve AEFI surveillance and documentations in Kebbi State and Nigeria, as a whole.

**Participant:** One, we need to select the qualified health workers first, let's talk about the quality and qualification, once the qualification is there and you have tested them and they have been certified to give injection, then you recruit them because we have so many health workers retiring and no fresh employment. As a result of no-fresh employment, you have a situation where availability becomes the desirability. What is available are those who have trained from the Colleges of Health Technology that were not licensed to give injection who will be next option to be retrained and to have access to injection. They (Environmental Health Officers and the likes) should be trained, they should be able to sensitize them on the issues such as the causes of AEFI. There are technical aspects, there are vaccine causation, there are other aspects (about nine) of it. But if they realise that the AEFI is not their personal fault then they will be act confidently to report and line list and document appropriately. Once they are not given the courage to report AEFI cases, if they have 10 cases, they will hardly report one case.

**Interviewer:** Which other issue, which other recommendation would you give?

**Participant:** Then, another recommendation is that the partners also should be coordinated in terms of AEFI surveillance. It is not a thing that only one organization should focus on. None of the organization should go for AEFI investigation without the other. Those that will be going for investigation should go with those with social mobilization, or C4D consultant, to manage the reality regarding of the profile of the child and the other aspect of sensitization for them (partners) to know that AEFI is not something that will kill the child. So, partners’ collaboration and coordination need to be there, because with partners’ collaboration and coordination there will be serious documentation. Once you investigate the society you will go on your surveillance and you find a lot of children then you will go back to the health facility as a team, can you give us the line list by settlements since now immunization is based on settlement once they are to bring then you will bring the investigation of the survey you have conducted then you compare. So once they see that there is tracking of performance and the rest of things, they also can be able to stand properly and do the right thing.

**Interviewer:** That is fine, anything else.

**Particpant:** Another thing is the remuneration is very very key for those RI providers. There remuneration should come also frequently to them.

Interviewer: Apart from that, any other things.

Participant: there is also, you need to reshuffle the staff

Interviewer: That is staff redistribution.

Participant: Yes, staff redistribution so that you can be able to compensate other RI providers

Interviewer: Areas where there is heavy workload, they should fix it

Participant: Yes, because some of the issue whereby you supposed to give something on the flesh and you are now giving it on the vein, maybe it can be caused because of stress on the RI providers

Interviewer: Okay, work overload can cause human error

Participant: Yes

Interviewer: That is fine, thank you. Any other thing, do you want to say anything anymore

Participant: I feel if the system is being properly managed, our RI will even be boosted very well.

Interviewer: If AEFI surveillance is effective and well managed and then the communication angle is if the data is used to generate communication strategy

Participant: Once it (AEFI surveillance) is effective, then it will give us a forge away ahead for community communication strategies

Interviewer: Then the other things I want to find out. Are you aware of the MED safety app

Participant: Med safety app, yes

Interviewer: You are aware

Participant: Yes, we have the Med safety app.

**Interviewer:** Do you think it can be integrated with the current system

**Participant:** Yes, of course.

**Interviewer:** Thank you very much, I am very grateful. But what do you think could be the problem with the MEDsafety app- its use in this country, particularly in a place like Kebbi State? What do you think could be challenge in the use of MED safety app to also report AEFI?

**Participant:** You know, something which is a new system, it will take a longer time at least for people to get into and report with it. But I think in the nearby future, it is the best.

**Interviewer:** Thank you very, am very grateful. Thanks for the opportunities and your time.
