# Supplementary material for: Barriers to optimal AEFI surveillance and documentation in Nigeria: Findings from a qualitative survey
Source: PLOS Glob Public Health. 2023 Sep 8;3(9):e0001658. doi: 10.1371/journal.pgph.0001658 (PMC10490937; doi:10.1371/journal.pgph.0001658)
Supplement: S1 Data — (ZIP) [file pgph.0001658.s002.zip › Transcription- interviews/WHO RI Focal Person.docx]

Interviewer: I will request your verbal consent to proceed with this discussion if you are comfortable with the study. You can please say go ahead and if otherwise we can stop the interview. Thank you. I am waiting for your response please.

Participant: Yes, we can proceed

Interviewer: Thank you very much for this opportunity. I hope it will benefit public health and humanity in general. The first question will try to elicit the capacity and the functionality of the AEFI surveillance system and we shall be using the CDC system evaluation attributes. So, I will ask a few questions. First of all, are you aware of the CDC surveillance system evaluation attributes.

Participant: Yes, am aware because I have read a while ago. Is talking about simplicity, flexibility, data quality and some things like that.

Interviewer: Is alright. Thank you very much. Do you think in your own experience or from what you know would you say that AEFI surveillance system in Nigeria is simple, is flexible, acceptable and sensitive enough to inform vaccine safety consideration. Would you say that?

Participant: Yes, I would say that to some extent because there are some issues where I can say it is not simple and then but majority of it is simple and flexible and then there is some level of data quality and acceptability.

Interviewer: Ok, I think, let me ask the question again because you have started talking about the next question, I want to ask you. Let me make it quite simple. Would you say that the AEFI surveillance system in Nigeria is simple and why if it is simple?

Participant: Well, I will say it is simple because the structure is clearly defined and then the reporting system also is clearly defined like from the health worker to the LGA DSNO. From LGA DSNO to the State Epidemiologist and then to the national level. So, I think the structure is simple because most of these people they are working together and in a clear manner and then the method of the reporting also is simple because it is paper based so and then the follow up that is being done in order for the system to continue, I think it is also and then it is like integrated in terms of not only routine immunization even during supplementary immunization activities and even other areas.

Interviewer: Will you say it is flexible

Participant: Yes, I would say it flexible

Interviewer: Base on what reason

Participant: Well, it is not really flexible because most of the reporting tools are hard copies so for you to change something it would be difficult. But if it is electronic, it will be more flexible. So and then if there is the use of technology advancement will improve flexibility but for now I don't think it is much flexible like that.

Interviewer: Do you think it is acceptable to the operators and also the sponsors and also the recipients of the product of the surveillance system. Do you think is acceptable.

Participant: Yes, I think it is acceptable but there are issues with the acceptability like some of the health workers they are afraid of reporting of AEFI because they are going to be penalized for reporting AEFI, thinking it is their fault or something like that. So, if there is no much enlightenment they may tend not to report more

Interviewer: So, how would you describe the level of acceptability then.

Participant: Well, it is good, I will say it is good but to some extent because some they accept it they can report but are reluctant to report thinking there may be consequences.

Interviewer: Would you say it is sensitive enough to inform vaccine safety consideration

Participant: Well yeah, I can say it is sensitive enough because like during any introduction of a new vaccine or any vaccine that there is a massive sensitization on AEFI. If it is routine immunization whenever the caregiver or the vaccinator gives you the vaccination you will ask to reveal anything that you are not comfortable to report when you come back (return visit). It is just that some caregivers are reluctant to report back.

Interviewer: Thank you very much. Do you think the data being generated from the AEFI surveillance system currently is of high quality. How would you describe the quality of data being generated?

Participant: Well, it is qualitative to my own perception but not of high quality. Yeah, it has some level of quality but not high because sometimes if you deep dive inside the forms and say you want to really see forms you will see the data is incomplete. So, there is incomplete entry and then even the timeliness sometimes is not and then even when they fill it some of the columns you will see that some do not even understand what really, they were asked to fill. So, I think there is some level and then even the quality of the training that the providers have on AEFI some do not really have that qualitative training to really generate a quality data.

Interviewer: So, overall based on what you have said, the above discussion would you say the current AEFI surveillance system is effective and robust enough to inform vaccine safety consideration

Participant: Yes

Interviewer: You are free to elaborate and you are free to use any adjective. I said based on the above discussion, would you say that the current AEFI surveillance is effective and robust enough to inform vaccine safety consideration?

Participant: Yes, I would say that it is effective and robust although it has some challenges and then some pros and cons too. So, it is effective but there are things that can be done to make it more effective and to improve upon what we are having now

Interviewer: So, do you think it is also robust enough or effective enough to inform or to develop communication strategy to inform demand generation for immunization

Participant: Yeah, I think it can be integrated so that it can inform demand creation because if you are assuring your clients that the vaccines are safe and then they don't have any AEFI so there would be, it would improve

Interviewer: So, in other words, are you saying that the data being generated could support public health system to develop communication strategy to inform demand generation for immunization

Participant: Yes, very well. Because if there are number of AEFI that are being generated then you can convince your clients that ok you can see there are no serious AEFI being generated from that vaccine. So, they would tend to come more for the vaccine that it would create more demand for the vaccine and then because they are assured it is safe.

Interviewer: That is fine. But as you have said earlier there might be need to improve certain areas like timeliness, data quality and supervision. That is fine. Thank you very much. Would you say that the surveillance system is stable?

Participant: Well, yeah. I will say it is stable in terms of is reliability and then the personnel and the data tools that are being used.

Interviewer: What are the challenges or bottlenecks impeding optimal AEFI surveillance and documentation in Nigeria based on your experience working in this environment-in Kebbi State?

Participant: I think there are many challenges in this area. You can have challenges in terms of coordination, in terms of reporting, in terms of data management and what have you. Like in terms of coordination, you see the AEFI committees are not very functional especially at the LGA level and at State level so but if you can strengthen these AEFI committees they can now give supportive supervision and then on-the-job-training and what have you to improve the AEFI surveillance system. And there is also under-reporting from holding of information by the health workers and then some caregivers they are not willing to report and then some of the caregivers are not well sensitised on how to or when to report because of work overload some of the vaccinators don't take their time to explain to the caregivers. Also, the poor capacity of health workers in terms of training and then on the job training and retraining, things like that. Then there is a reluctance giving feedback on the AEFI at least if you have something you are supposed to be given feedback. And then even availability of data tools, some data tools are not really available. Then lack of timely analysis that some of the focal points they don't do timely analysis and what have you. There is incomplete documentation sometimes on the data tools and the incorrect data entry and then the forms are not being checked regularly to ensure that they are being completely filled. So, I think there are many challenges in terms of AEFI surveillance system in Nigeria.

Interviewer: What is your perception regarding the functionality of AEFI surveillance and documentation for routine immunisation compared to supplementary immunization activities or OBR?

Participant: Well, my perception is that considering the data that we used to generate, I think we would say that of supplementary immunization activities is more effective because you will see more reports coming and is just because it is in a very short time that you will do supplementary immunization activities so there will be a lot of sensitization and then a lot of people are being vaccinated over a short period of time. So, and the reporting could be easily done. But for routine immunization as the name implies, it is routine and it is over a very large period and it is done may be in some health facilities but this one is in a campaign. In health facilities you tend to have more workload and you have other activities to do. So, you may not report quality data and then it may be incomplete, you may even under-report because you are may be alone manning all other activities in the health facilities. So, the data quality in terms of the routine immunization part is less than that in the supplementary immunization activities. The channel of reporting even in supplementary Immunization activities- the ward focal persons are involved- they would get all the data at a time and then they would submit to the DSNO and then from the LGA DSNO to the State and what have you but during the routine immunization they would wait until the end of the month then if you report then that is when they will collect and if they are reluctant to report you may just put zero and there would be no (case of AEFI) report. But if you can strengthen the system by improving the supportive supervision and then making the data tools may be electronic and user-friendly than these hard copies that we are using I think it can improve the system and then the quality of training and retraining and on-the-job supportive supervision. I think it can improve.

Interviewer: I want to know about AEFI reporting and documentation at the facility level. Also, how it feeds into LGA level and how the LGA level data feeds into IDSR 003 or the IDSR system and the DHIS 2. So how would you describe AEF surveillance and documentation at the health facility level in the State.

Participant: At the health facility level, I think the AEFI surveillance system in terms of documentation, it is fairly-documented because you will see the forms. They have like three or four forms to fill. Some will be reluctant to fill all of the line listing, the monthly reporting forms, the investigation forms and what have you. So, there is a tendency that the data could be incomplete and then timely submission also they will be reluctant to timely submit it to the LGA level but at the LGA level, I think whatever data they have they do transmit the complete data to the state level and other higher levels and then if it is a serious AEFI I know they do call they communicate directly using phone or whatsapp just to inform that ok there is a serious AEFI to come to investigate although is very rare for you to see serious AEFI in terms of documentation.

Interviewer: So, in two, three sentences, how would you describe that surveillance and documentation at the health facility. And in one sentence how would you describe it in terms of the functionality in one sentence how would you describe it for AEFI surveillance and documentation

Participant: Yeah, it is still functional but not to the extent that an ideal system should be. Yeah, it is functional because there is a level of reporting ongoing and then documentation

Interviewer: Do you mean not optimal, functional but not optimal?

Participant: Yes, not optimal. It is functional but not optimal.

Interviewer: How would you describe the reporting system and data transmission to the LGA?

Participant: Yeah, that is what I earlier said. The reporting system is manual- paper-based. So, there is incomplete reporting and the timeliness also is not very good, honestly. So, the completeness and the timeliness are not optimal.

Interviewer: What about the data quality?

Participant: Well, the data quality also is not optimal

Interviewer: Thank you, based on your experience, how would you describe the LGA level AEFI data linkage with the existing data management platform particularly IDSR 003, DHIS2, SORMAS and the likes. So, how would you describe the linkage that LGA level data with those data management platforms?

Participant: I would say it is poor. There is poor linkage in them because if you go at the LGA level you will see the hard copies of the line list and what have you, dumped there. But if you cross checking the DHIS 2 the number will not tally may be on the hard copy you will see 100 AEFI cases reluctancy of inputting it into DHIS system or other electronic system you will see may be only ten out of 100 that were recorded. So, there is a huge disparity honestly. So there is no proper linkage but if you can strengthen the linkage and ensure that whatever we have in the hard copy that was submitted from the heath facilities is also being recorded in the electronic platform or if we can generate electronically since from the health facility so that it will be transmitted directly to the LGA and all levels. I think it will capture more cases than leaving it at the paper based and then inputting it at the LGA

Interviewer: So, based on your level of technical competence, your knowledge and your experience working in this field what will be the recommendations that you will offer or suggest to improve or to achieve optimal AEFI surveillance and documentation in Kebbi State and Nigeria in general.

Participant: Well, I think there are many things actually like in terms of coordination we can reactivate the State and LGA AEFI committees to make them functional so that they will oversee all the AEFI activities at the LGA level and State level and then let us ensure that we establish AEFI focal point in all the health facilities so that they will be sensitizing the other health workers and reminding them of reporting. And then giving them (health workers) on-the-job-training and then by strengthening the collaboration between the DSNOs and the LIOs and routine immunization officers at the LGA level so that they will strengthen the linkage. And then the reporting will improve and then encouraging the health workers to be reporting and assuring them that there is no penalty for reporting any AEFI and then RI service providers should also be sensitized and should be reminded to sensitize parents and caregivers to be reporting AEFI to their respective vaccinators. And then community sensitisation of community informants and other community resources on AEFI can also improve on that. Then in terms of capacity and management, I think training and retraining of the healthcare workers on AEFI surveillance will also improve the system and then on-the-job-training also of the healthcare workers should also improve. Then the reluctance in investigation of AEFI - the LGAs should ensure timely investigation of all reported serious cases so that whenever a health worker report AEFI, they should do prompt investigation and then giving feedback is very important and then if we can improve feedback mechanism that whenever you report an AEFI may be it serious or not serious we need feedback from higher level. Timely analysis of the AEFI data and then giving feedback to the health workers and caregivers. And then on data quality also and reporting, if we can make the data reporting system to be electronic like using smart phones. I think it will be more accurate and it will be more timely, and then it will be more complete rather than using the paper-based and then if the forms may be the information can be summarized-maybe the lengthy forms are what is scaring some of our healthcare workers on filling it. I think if we can do that and then we do regular supportive supervision to the health facilities and then may be doing like monthly meetings on AEFI or quarterly meetings discussing on AEFI challenges and how to overcome those challenges, I think then even sensitization of the traditional leaders I think they can also help because some may be reporting to them if they don't know what to do, they may not report to the health facility. So, I think these are some of the things that we can do to improve AEFi surveillance system in Kebbi State and Nigeria.

Interviewer: Thank you very much. Thank you for your time and the opportunity to learn from your experience to improve public health AEFI surveillance particularly in Kebbi State and Nigeria as a whole. We hope that, God-willing, the findings of this research will be published and will be assessable to the academia and public health practitioner. Then we disseminate it so that it will be to able inform policy and practice. Thank you once again

Participant: You are welcome
