# Supplementary material for: Barriers to optimal AEFI surveillance and documentation in Nigeria: Findings from a qualitative survey
Source: PLOS Glob Public Health. 2023 Sep 8;3(9):e0001658. doi: 10.1371/journal.pgph.0001658 (PMC10490937; doi:10.1371/journal.pgph.0001658)
Supplement: Attachment — Submitted filename: Responses to Reviewers Comments- PGPH-D-22-01497.docx [file pgph.0001658.s007.docx]

**Date: 9^th^ December 2022**

**To: Dr Peter Bai James, The Academic Editor, PLOS Global Public Health (PGPH)**

**From: Authors**

Re: "Barriers to Optimal AEFI Surveillance and Documentation in Nigeria: Findings from a Qualitative Survey**”** (PGPH-D-22-01497)

Thank you for coordinating the review of our article. The opportunity to revise the manuscript is well appreciated. Corrections have also been effected in the revised manuscript.

**Journal Requirements:**

1. In the online submission form, you indicated that "The recordings and transcripts for this work are available upon request to the corresponding author.". All PLOS journals now require all data underlying the findings described in their manuscript to be freely available to other researchers, either 1. In a public repository, 2. Within the manuscript itself, or 3. Uploaded as supplementary information. This policy applies to all data except where public deposition would breach compliance with the protocol approved by your research ethics board. If your data cannot be made publicly available for ethical or legal reasons (e.g., public availability would compromise patient privacy), please explain your reasons by return email and your exemption request will be escalated to the editor for approval. Your exemption request will be handled independently and will not hold up the peer review process, but will need to be resolved should your manuscript be accepted for publication. One of the Editorial team will then be in touch if there are any issues.

**Authors’ Response:** The journal requirement was well-noted. The file has been duly uploaded.

**Additional Editor Comments (if provided):**
1. The authors need to explain how the trustworthiness of the study was i.e., Credibility, Dependability, Confirmability and Transferability.

**Authors’ Response:** Thanks for the inquiry. This qualitative research study is trustworthy based on the following (using the criteria such as credibility, dependability e.t.c):

***Credibility:*** The principal investigator had sustained engagements with key stakeholders in AEFI surveillance and Immunisation space within the study setting through IDI and FGD sessions. The study research questions were based on a decade of professional work experience as a development partner and routine AEFI data collection. Hence, the likelihood of misinterpretation and misrepresentation of findings and data is almost nil. Further, the findings had undergone peer review within the faculty also to ascertain the credibility of the process and findings.

***Transferability:*** This refers to the generalizability of the inquiry. The methods of investigation were clear, and the categories and number of participants and bases for the purposive selection were detailed in the manuscript. This could be plausible in settings with similar structures and reporting systems. The findings are also transferable given the similarities in the underlying health system factors in-country and national stakeholders’ perspectives. This largely confers the generalizability of the study findings.

***Dependability:*** The author made effort to achieve dependability by ensuring that the research process is clear, replicable and well-documented. The recordings have been provided for transparency and potential audit purposes.

***Confirmability:*** According to Gulba and Lincoln (1989), confirmability is established when the researcher can demonstrate credibility, dependability and transferability. Essentially, it has to do with demonstrating how the researchers’ interpretation and findings are derived from the data. In this study, the authors have demonstrated these criteria.

2. The authors should ensure that the COREQ guidelines for reporting qualitative study is adhered to. Please submit a supplementary file of COREQ guidelines, which shows that the study adhered to the guideline.

**Authors’ Response:** Revision made in adherence to the COREQ guidelines. A completed COREQ guideline for reporting qualitative study has been provided as a supplementary file.

3. The authors need to state who conducted the interviews and analyzed, including his/her competency

**Authors’ Response:** The principal investigator (corresponding author) conducted the interviews and analyses. This was vetted by co-investigators. The corresponding author conducted and published previous qualitative studies in reputable journals such as BMC Public Health and BMC Health Services Research.

4. Can the authors provide basic demographics of the participants for the FGD and IDI?

**Authors’ Response:** This has now been supplied, please find attached as a supplementary document.

 5. Line 156. Please remove the z

**Authors’ Response:** This has been removed. Thank you.

**Comments to the Author**

1. Does this manuscript meet PLOS Global Public Health’s [publication criteria](https://journals.plos.org/globalpublichealth/s/criteria-for-publication)? Is the manuscript technically sound, and do the data support the conclusions? The manuscript must describe methodologically and ethically rigorous research with conclusions that are appropriately drawn based on the data presented.

Reviewer #1: Yes

Reviewer #2: Yes

2. Has the statistical analysis been performed appropriately and rigorously?

Reviewer #1: N/A

Reviewer #2: Yes

3. Have the authors made all data underlying the findings in their manuscript fully available (please refer to the Data Availability Statement at the start of the manuscript PDF file)?

The [PLOS Data policy](https://journals.plos.org/globalpublichealth/s/data-availability) requires authors to make all data underlying the findings described in their manuscript fully available without restriction, with rare exception. The data should be provided as part of the manuscript or its supporting information, or deposited to a public repository. For example, in addition to summary statistics, the data points behind means, medians and variance measures should be available. If there are restrictions on publicly sharing data—e.g. participant privacy or use of data from a third party—those must be specified.

Reviewer #1: No

Reviewer #2: No

4. Is the manuscript presented in an intelligible fashion and written in standard English?

PLOS Global Public Health does not copyedit accepted manuscripts, so the language in submitted articles must be clear, correct, and unambiguous. Any typographical or grammatical errors should be corrected at revision, so please note any specific errors here.

Reviewer #1: Yes

Reviewer #2: Yes

5. Review Comments to the Author

Please use the space provided to explain your answers to the questions above. You may also include additional comments for the author, including concerns about dual publication, research ethics, or publication ethics. (Please upload your review as an attachment if it exceeds 20,000 characters)

**Reviewer 1: Comments**

Reviewer #1: This is a very interesting paper that identifies very real and practical challenges to AEFI surveillance in low resource settings.

The introduction appropriately places the problem in context.

The methods are generally sound.

The results are compelling and of interest.

The discussion places the findings in context of existing research appropriately demonstrating general consistency of the findings.

**Response:** Thanks for the compliments and positive remarks on the quality and value of the manuscript to the subject of AEFI surveillance in low resource settings.

My comments:

1. The sample size is small - this would be the main limitation of the paper. The potential sample however is limited.

**Authors’ response:** Thanks for the comments. The sample size may not be considerably different from this, given the number of relevant informants who are competent and positioned to provide well-informed or expert opinions, especially at the subnational level. This was alluded to by the reviewer. Beyond this, the concept of saturation guided the authors’ decision not to consider exploring other potential informants.

2. It wasn’t clear how the interview guide was constructed. More information on this would be worthwhile.

**Authors’ response:** Thanks for the suggestion. A subsection of the interview guide has been included. The interview guide was constructed from the research objectives and the existing literature on the subject of investigation.

3. I don’t believe any comment was made on whether saturation of themes was achieved.

**Authors’ response:** Thanks for the observation. Comments on the attainment of saturation have been included in the revised manuscript.

4. For the results the quotes may work best in a results table.

**Authors’ response:** Thank you. This has been done accordingly in the revised manuscript.

5. For the discussion - while the results are placed in the LMIC context I dont believe WHO's extensive guidance on AEFI reporting is referenced. https://www.who.int/teams/regulation-prequalification/regulation-and-safety/pharmacovigilance/health-professionals-info/aefi
It seems like the themes identified in this and other papers referenced would be relevant to the WHO.

**Author’s response:** Similar and relevant WHO documents have been cited in the manuscript (please see the reference list)

6, A brief mention of digital reporting of AEFI reporting was alluded to - some more description on this would be interesting.
**Authors’ response:** Noted with thanks. Additional details have been provided: ***“****Med Safety App will improve AEFI cases detection, reporting and documentation. The full deployment of Med Safety App will permit real-time analysis and feedback. It permits flexibility (for new vaccine surveillance such as COVID-19) and self-reporting, among other health information and surveillance system strengthening benefits.”*

7. It wasn’t clear to me how AEFI data is used in Nigeria. Some information on data flows, and processes for handling and responding to AEFI data would be helpful. I think other sections of the discussion could be reduced to allow for this.

**Authors’ Response:** A brief on the reporting system and data flow has been included in the revised version of the manuscript (pages 5 and 6)

**Reviewer #2: Comments**

**Abstract**
- The introduction should highlight the global burden of AEFI to set the tone for the relevance of the study.

**Authors’ response:** Thanks for the feedback. This has been addressed in the revised manuscript.

**Reviewer-** The significance of the study needs to be well established. Indicating that the performance remains weak does not present the clear problem/justification of the study.

**Authors’ response:** Thanks for the comments. The author has provided justification for the study (see please revised abstract)

**Reviewer -** The study is a qualitative design, however, the type of qualitative design was not stated (phenomenological, ethnographic, grounded theory, historical, case study, and action research etc)

**Authors’ response:** Grounded theory approach was used in the qualitative study design.

**Reviewer-** Public health action from the study was not indicated which makes which underscores the relevance of the study

**Authors’ response:** This has been adequately addressed in the revised abstract. Thank you.

**Introduction**
**Reviewer-** Line 72 - 74: the objectives are not clear. It should be measurable. The authors should conduct literature review to understand what the quantitative studies found. Since they claim no qualitative study has been conducted, this study should rather help in exploring the findings from the quantitative studies.

**Authors’ response:** Thanks for the comments. This has been addressed in the revised version of the manuscript. A clear objective has been stated and evidence of a gap in knowledge based on the literature review has been demonstrated in the revised version.

**Reviewer-** Line 106: Design should reflect in abstract

**Authors’ response:** This has been duly addressed in the abstract section of the revised manuscript.

**Reviewer-** Line 123: What informed the sample size for the FGD?

**Authors’ response:** Thanks for the comment. Given that we used the grounded theory approach, we purposively selected the participants and sample size determination was guided by the existing secondary data and the concept of saturation.

**Reviewer-** Line 127: Spell out in full for first use: NAFDAC and other abbreviations

**Authors’ response:** NAFDAC and other abbreviations have been spelt out for the first use.

**Methods**
Reviewer- The methods should be aligned to the objectives of the study with sub-headings (sub-themes) for each objective. A it stands the objectives are equally not clear which makes it difficult to appreciate the linkages between the objectives and the methods.

**Authors’ response:** The objective has been clearly stated (as previously requested) and the method section revised to improve clarity. Thank you.

**Results**
It would be good to have a table summarising the positive and negative sentiments to help the reader appreciate the findings in a snapshot.

**Authors’ response:** Thanks for the suggestion, it was well-taken. A table showing the summary of the key results from the interviews and FGD has been included in the revised manuscript.

**Discussion**
**Reviewer-** Sequence should be revised to follow the presentation of results to provide more clarity.

**Authors’ response:** Some adjustments have been made to follow the presentation of the results. Thank you.

**Reviewer** -Line 423: the word "gap" should be removed. The word inadequate is enough to indicate a negative direction of communication.

**Authors’ response:** The tautology has been corrected. The word “gap” has been removed.

**Reviewer-** Authors should indicate the limitations of the study

**Authors’ response:** The limitations have been included in the revised manuscript.

**Conclusion**
- Authors did not indicate the public health actions the emanated from their work which underscores the relevance of the study.

**Authors’ response:** The conclusion has undergone a major revision. Please see the revised manuscript. Thank you.

**Reviewer-** the authors talked about addressing the challenges identified, however there are not targeted recommendations for their work and no evidence to show that their study was of any benefit to the study population

**Authors’ response:** Thanks for the useful comments. This has been adequately addressed in the revised version.

Finally, we thank the reviewers for their constructive feedback, which improved the manuscript's quality.
