# Supplementary material for: Barriers to optimal AEFI surveillance and documentation in Nigeria: Findings from a qualitative survey
Source: PLOS Glob Public Health. 2023 Sep 8;3(9):e0001658. doi: 10.1371/journal.pgph.0001658 (PMC10490937; doi:10.1371/journal.pgph.0001658)
Supplement: Attachment — Submitted filename: Responses to Reviewers Comments- PGPH-D-22-01497 R2.docx [file pgph.0001658.s008.docx]

**Date: 7^th^ February 2023**

**To: Dr Peter Bai James, The Academic Editor, PLOS Global Public Health (PGPH)**

**From: Authors**

Re: "Barriers to Optimal AEFI Surveillance and Documentation in Nigeria: Findings from a Qualitative Survey**”** (PGPH-D-22-01497)

Thank you for coordinating the review of our article. The opportunity to undertake a minor revision is appreciated. Corrections have also been effected in the revised manuscript.

**EDITOR Comments:**

1. In the results sections, I suggest that you reduce some of the quotes to just one or two representative quotes.

2. Please briefly describe how trustworthiness of the findings was ensured, i.e.  how credibility, dependability, confirmability and transferability was ensured when conducting this study.

**Authors’ response:** Thanks for the suggestion. It has been effected and incorporated in the revised manuscript. Please see pages 12-13 in the revised manuscript.

3. Please upload a supplementary file that indicate that you adhered to the COREQ guidelines for qualitative research.

**Authors’ Response:** The revised manuscript adhered to the COREQ guidelines. A completed COREQ guideline for reporting qualitative study was provided as a supplementary file during the initial round of revision. Thank you.

**Reviewer #1:** The authors have satsifactorily addressed my comments. I would still remove at least some of the quotes from the results as some of them are also in a table. The paper does seem long and the discussion in particular. The issue of fear of reporting AEFI's seem to me the most important finding and its not addressed in the recommendations.

**Authors’ response:** As suggested, the quotes from the results have been trimmed. The fear of reporting AEFI is now reflected in the recommendations (please see the revised manuscript). Thank you.

**Reviewer #2:** Data should be uploaded.

**Authors’ response:** This has been addressed in the initial round of review. Thank you.

Finally, we thank the reviewers and the editor for their constructive feedback, improving the manuscript's quality.
